# The Relationship Between Adherence to the Mediterranean Diet, Oxidative Stress, Trimethylamine N-Oxide, and Inflammatory Markers in Patients with Metabolic Dysfunction-Associated Steatotic Liver Disease

**DOI:** 10.3390/nu18142231

**Published:** 2026-07-09

**Authors:** Saibe Merve Kazdal, Medeni Arpa, Çağlayan Keklikkiran, Sevinç Yücecan

**Affiliations:** 1 Department of Nutrition and Dietetics, Institute of Health Sciences, Lokman Hekim University, 06510 Ankara, Türkiye; sevinc.yucecan@lokmanhekim.edu.tr; 2 Department of Medical Biochemistry, Faculty of Medicine, Recep Tayyip Erdogan University, 53100 Rize, Türkiye; medeni.arpa@erdogan.edu.tr; 3 Department of Gastroenterology, Faculty of Medicine, Recep Tayyip Erdogan University, 53100 Rize, Türkiye; caglayan.keklikkiran@erdogan.edu.tr

**Keywords:** MASLD, FibroScan, IPAQ-SF, MEDAS, TMAO, oxidative stress, inflammation, Mediterranean diet

## Abstract

**Background/Objectives**: The aim of this study is to evaluate the relationship between adherence to the Mediterranean diet and intestinal microbiota metabolite trimethylamine N-oxide (TMAO) levels, systemic inflammation markers, and oxidative stress parameters in patients with metabolic dysfunction-associated steatotic liver disease (MASLD). **Methods**: MASLD patients whose fatty liver and fibrosis severity were determined with FibroScan and a healthy control group were included in the study. In addition to the routine biochemical parameters, serum TMAO levels, inflammatory cytokines (IL-6, IL-10, TNF-α), and oxidative stress indicators (malondialdehyde, glutathione, and MDA/GSH ratio) of all participants were measured. In comparing these parameters between fibrosis groups, adjustment for potential confounding variables (age, BMI, sex, comorbidity, relevant medication use, MEDAS score, and physical activity) was performed. Following anthropometric evaluations, the adherence levels of the participants to the Mediterranean diet were determined by the Mediterranean Diet Adherence Screening Scale (MEDAS), and their physical activity levels were determined through the International Physical Activity Questionnaire-Short Form (IPAQ-SF). **Results:** Individuals diagnosed with MASLD had significantly lower adherence to the Mediterranean diet and lower physical activity levels compared with healthy controls. Among the inflammatory parameters, IL-6 levels were significantly higher in the advanced fibrosis group than in the early-stage and significant fibrosis groups, while TNF-α levels were significantly higher in the advanced fibrosis group than in the early fibrosis group. No significant differences were observed among the groups in TMAO, IL-10, GSH levels, or the MDA/GSH ratio; however, among the oxidative stress markers, MDA levels were significantly higher in the early fibrosis group than in the significant fibrosis group. **Conclusions:** In MASLD patients, fibrosis severity was associated with alterations in TMAO, inflammatory, and oxidative stress markers, with increased TNF-α and IL-6 levels reflecting a high inflammatory burden as fibrosis progressed. Lower TMAO levels in fibrosis groups likely reflect reduced hepatic FMO3 activity, lower fish intake, and medication use, while elevated MDA levels in early-stage fibrosis suggest that lipid peroxidation may be more prominent in the initial phases of disease. Mediterranean diet adherence and physical activity were inversely associated with pro-inflammatory markers and MASLD severity, being highest in the control group, suggesting a potential protective role for these lifestyle factors in MASLD progression.

## 1. Introduction

The term non-alcoholic fatty liver disease (NAFLD) was renamed as metabolic dysfunction-associated steatotic liver disease (MASLD) with the multicenter Delphi consensus paper published in 2023 [1]. Metabolic dysfunction-associated steatotic liver disease (MASLD) is defined as a fatty liver accompanying impaired metabolic status. MASLD is characterized by the accumulation of excess fat in the liver. It is often associated with many conditions, including obesity, hypertension, diabetes, insulin resistance, metabolic syndrome, inflammatory responses, and the hepato-gut axis of metabolic dysfunction [2,3]. Although it was initially considered a benign liver disease, current evidence has shown that it can also develop in individuals without a history of alcohol consumption due to genetic and environmental factors and can lead to fatty liver (steatohepatitis), fibrosis (hardening of liver tissue), and cirrhosis if left untreated [2,4,5].

Currently, liver biopsy is known to be the most reliable method for the traditional diagnosis and histological evaluation (steatosis and fibrosis) of MASLD and is considered the gold standard. However, the fact that biopsy is not used in the early stages of a potential patient’s diagnostic journey, together with its invasive nature and its high cost, makes it unsuitable for routine screening or monitoring [6,7]. When evaluating non-invasive diagnostic biomarkers and tools to assess the severity and progression of MASLD, FibroScan is a widely used non-invasive imaging tool to bridge the gap between accessibility and quantification, measuring liver stiffness (liver stiffness measurement [LSM]) and hepatic fat content (controlled attenuation parameter [CAP]) [8]. Different liver fibrosis staging approaches based on the Metavir scoring system were combined under a single standard classification system as no fibrosis (F0), mild fibrosis (≥F1), significant fibrosis (≥F2), advanced fibrosis (≥F3), and cirrhosis (F4) [9]. Since the fibrosis stage is the main determinant of prognosis, proper assessment is essential for risk classification, treatment decision-making, and longitudinal monitoring of disease progression or remission [10].

The main treatment of MASLD is lifestyle change consisting of diet, activity, exercise, and weight loss. The Mediterranean diet is the most studied diet model in the management of MASLD and has been included in the literature by conducted studies as an effective nutritional method in its treatment [4,11,12]. Inflammation is a significant factor in the progression of MASLD, and the Mediterranean diet has a protective and enhancing effect on health, reducing the risk of diet-related chronic diseases. It is also suggested that, due to the harmonious combination of many foods with anti-fibrotic, anti-inflammatory, and anti-oxidative components, it represents one of the healthiest dietary patterns in preventive medicine and can provide health benefits, including reducing liver inflammation [13,14]. These health benefits are linked to the effects of antioxidants such as tocopherol, carotene, and ascorbic acid, as well as flavonoids like polyphenols and anthocyanins, which are components of the Mediterranean diet [15]. In addition, physical activity, together with diet, is a key lifestyle component in the prevention and management of MASLD, and since it is closely associated with insulin resistance, visceral adiposity, dyslipidemia, and chronic low-grade inflammation, decreased physical activity levels and a sedentary lifestyle can increase the metabolic burden of the disease [16,17].

Trimethylamine N-oxide (TMAO) is a metabolite of choline and L-carnitine-rich foods such as red meat, resulting from the fermentation of dietary choline, carnitine, and betaine by gut bacteria to produce TMA, which is further oxidized by liver flavin monooxygenases (FMO3) and converted into TMAO [18,19]. TMAO has recently been considered a candidate risk factor for many chronic diseases, including cardiovascular disease, kidney disease, type 2 diabetes mellitus, and MASLD [20,21].

The pathogenesis of MASLD is shaped by the interaction of complex and multifaceted pathophysiological mechanisms. In this process, inflammation, oxidative stress, and endothelial dysfunction play critical roles ranging from the onset of the disease to its progression [22]. Although the traditional “two-stroke” hypothesis has long been accepted in explaining the pathogenesis of MASLD, current research has revealed that this model is inadequate, leading to a paradigm shift towards the theory of “multiple parallel strokes.” This current approach highlights the simultaneous and synergistic interaction of multiple factors, such as insulin resistance, dietary habits, intestinal endotoxemia, genetic predisposition, and epigenetic modifications, which shape the spectrum of disease progression from simple hepatic steatosis to MASH and fibrosis. Oxidative stress is considered the main factor that combines all of the above-mentioned pathogenic factors and leads to hepatocellular death and tissue damage [23]. In the case of chronic energy excess, exceeding the triglyceride storage capacity of the liver triggers free fatty acid accumulation in hepatocytes, which leads to mitochondrial dysfunction. Mitochondrial dysfunction causes overproduction of reactive oxygen species (ROS), disrupting oxidative balance and increasing cellular damage. This process, coupled with the activation of lipotoxic lipid metabolites and the triggering of pro-inflammatory pathways, plays a key role in the progression of MASLD from simple steatosis to inflammatory and fibrotic stages [24,25]. Therefore, understanding the role of oxidative stress and inflammation in the pathogenesis of MASLD is critical in developing strategies for disease prevention and treatment.

Conventional ultrasound is widely used in clinical practice to detect hepatic steatosis; however, it is not adequate for accurately staging fibrosis [26]. For this reason, current guidelines recommend non-invasive elastography methods such as FibroScan (vibration-controlled transient elastography, VCTE) [16]. Accordingly, the use of FibroScan in the present study represents an important methodological strength of this work [27].

Although previous studies have examined Mediterranean diet adherence, physical activity levels, TMAO, inflammatory biomarkers, and oxidative stress parameters in patients with MASLD, these variables have generally been evaluated in isolation. The present study, by contrast, assessed these parameters collectively within a single cohort. A particular strength of our design is the balanced representation of patients with advanced fibrosis/cirrhosis (F3–F4) alongside the control, early-stage fibrosis, and significant-stage fibrosis groups, which allowed Mediterranean diet adherence, TMAO levels, inflammatory biomarkers, and oxidative stress markers to be evaluated comprehensively across the entire spectrum of liver fibrosis severity. The Mediterranean diet is a well-established dietary pattern recognized for its anti-inflammatory and hepatoprotective properties [28,29]. In this study, we hypothesized that adherence to this dietary pattern would progressively decrease with increasing fibrosis severity, and that this decline would be paralleled by corresponding alterations in inflammatory biomarkers, oxidative stress parameters, and TMAO levels across the entire spectrum of liver fibrosis, from early stage to advanced disease.

## 2. Materials and Methods

### 2.1. Experimental Approach

This cross-sectional descriptive study was conducted with patients diagnosed with MASLD (study group) who presented to the Gastroenterology outpatient clinic of Recep Tayyip Erdoğan University (RTEU) Training and Research Hospital between March 2025 and November 2025, and individuals who applied to the outpatient clinics who met the criteria of the control group (control group). The study was conducted in accordance with the principles of the Declaration of Helsinki. Ethics committee permission was obtained from Lokman Hekim University Non-Interventional Clinical Research Ethics Committee (Decision No: 2025/27-Code No: 2025013).

### 2.2. Data Collection Approach

Inclusion Criteria: Patients over 18 years of age diagnosed with MASLD who presented to the Gastroenterology outpatient clinic of RTEU and Research Hospital, and a control group of individuals without any liver disease other than hemangioma who presented to the outpatient clinics of RTEU Training and Research Hospital were included in the study. According to the 2024 EASL–EASD–EASO Clinical Practice Guidelines, a diagnosis of MASLD requires the presence of hepatic steatosis and at least one cardiometabolic risk factor, excluding other causes of hepatic steatosis. Cardiometabolic risk factors, as reported in the literature, include: body mass index (BMI) > 25 kg/m^2^ or waist circumference > 94 cm in men, >80 cm in women; fasting blood glucose ≥ 100 mg/dL or 2 h post-oral glucose tolerance test (OGTT) blood glucose ≥ 140 mg/dL or HbA1c ≥ 5.7% or Type 2 Diabetes Mellitus (T2DM); blood pressure ≥ 130/85 mm/Hg or receiving antihypertensive treatment; plasma triglyceride level ≥ 150 mg/dL or receiving lipid-lowering treatment; plasma HDL cholesterol ≤ 40 mg/dL in men, ≤50 mg/dL in women or receiving lipid-lowering treatment [16,30].

Exclusion Criteria: Patients with hepatitis B virus, hepatitis C virus, autoimmune hepatitis, fatty liver disease not associated with metabolic dysfunction, hypopituitarism, primary or secondary Cushing’s syndrome, inflammatory bowel disease, active cancer or a cancer diagnosis and treatment within the last two years, celiac disease, alpha-1-antitrypsin deficiency, gastric or small bowel surgery that may lead to malabsorption, drug-induced fatty liver disease (corticosteroids, amiodarone, ibuprofen, valproic acid, perhexiline, anabolic steroids, methotrexate, tamoxifen, aspirin, cisplatin, and irinotecan), voluntary or involuntary rapid weight loss, excessive alcohol consumption (≥20 and ≥30 g per day for women and men respectively), use of dietary supplements and herbal products that may affect liver function (artichoke extract, milk thistle extract, etc.), substance abuse, and patients considered to have potential cooperation problems for the study were excluded.

### 2.3. FibroScan

Before being referred from the gastroenterology outpatient clinic to the dietetics outpatient clinic, routine blood tests were requested from the participants in the study group, and liver stiffness/fibrosis (LSM) and liver steatosis/fatty liver (CAP) were evaluated using vibration-controlled transient elastography (VCTE; FibroScan 630 Expert, Echosens SA, Paris, France dual-probe machine). Blood samples were taken from participants after at least 8 h of fasting, and FibroScan tests were performed by a gastroenterologist. The procedure was performed in the supine position using the Echosense dual-probe machine (medium or X-large probes were used, depending on whether the subcutaneous fat accumulation was less than or greater than 2.5 cm). Based on the FibroScan test, fatty liver disease and fibrosis will be graded. Regarding FibroScan cut-offs, the measurements consist of the following: S0: <238 dB/m, S1: 238–258 dB/m, S2: 259–299 dB/m, S3: ≥300 dB/m (decibels per meter), F0: ≤6.0 kPa, F1: 6.1–7.9 kPa, F2: 8.0–9.9 kPa, F3: 10.0–13.49 kPa, F4: ≥13.5 kPa (kiloPascal).

### 2.4. Anthropometric Measurements

Participants underwent height, neck circumference, waist circumference, hip circumference, body weight measurement, and body analysis. BMI is calculated as weight (kg)/height (m^2^). During body weight measurement and body analysis, body fat percentage (%), fat and muscle mass (kg), and visceral fat level were recorded using bioelectrical impedance analysis (BIA) with a Tanita BC-420 MA (Tanita Corp., Tokyo, Japan) device. A minimum fasting period of 3 h was required for the BIA, and individuals deemed unsuitable for the analysis were excluded from the study.

Participants’ heights were measured barefoot with a rigid measuring tape, with their feet together and against a wall, their heads in the Frankfurt plane, and their eye triangle and the tops of their ears aligned [31]. Neck circumference measurement is used as an indicator of upper-body fat distribution [32]. Waist circumference measurement indicates abdominal fat and organ fat distribution. A waist circumference of ≥94 cm in men and ≥80 cm in women is considered a risk factor [32]. Waist-to-hip ratio is a key indicator of abdominal obesity, and according to World Health Organization criteria, values above 0.85 in women and 0.90 in men are associated with increased cardiometabolic risk [33].

### 2.5. MEDAS (Mediterranean Diet Adherence Screening Scale)

The study used MEDAS, an internationally recognized scale, to assess diet quality. The MEDAS scale consists of a total of 14 questions. The MEDAS scale, the validity and reliability of which have been verified by Pehlivanoğlu et al., was used [34]. A total score of 7 or higher indicates that the individual has an acceptable degree of adherence/compliance with the Mediterranean diet. A total score of 9 or higher indicates that the individual is strictly adhering to the Mediterranean diet. As the scale score increases, adherence/compliance with the Mediterranean diet increases. In the scale evaluation, a score of 0–6 indicates inconsistency, 7–8 indicates agreement, and 9–14 indicates close agreement.

### 2.6. IPAQ-SF (International Physical Activity Questionnaire-Short Form)

The International Physical Activity Questionnaire-Short Form provides information on time spent walking, engaging in moderate and vigorous activity, and sitting. The short form’s total score is calculated as the sum of the duration (minutes) and frequency (days) of walking, moderate-intensity activity, and vigorous activity. The energy required for activities is calculated using the MET-minute score. The validity and reliability of the relevant questionnaire in Turkish were established by Öztürk et al. [17]. The IPAQ-SF total physical activity score was calculated in MET-min/week, and individuals were classified as inactive (<600 MET-min/week), minimally active/insufficient physical activity (600–3000 MET-min/week), and very active/sufficient physical activity (>3000 MET-min/week).

### 2.7. Biochemical Parameters

Biochemical parameters were obtained from routine laboratory tests performed at the Medical Biochemistry Laboratory of RTEU Training and Research Hospital. The following parameters were recorded: fasting blood glucose (FBG), total cholesterol (TC), high-density lipoprotein cholesterol (HDL-C), low-density lipoprotein cholesterol (LDL-C), triglycerides (TG), alanine aminotransferase (ALT), aspartate aminotransferase (AST), gamma-glutamyl transferase (GGT), ferritin, albumin, uric acid (UA), C-reactive protein (CRP), insulin, hemoglobin A1c (HbA1c), lymphocyte count (LYM), platelet count (PLT), direct bilirubin (DBil), total bilirubin (TBil), homeostasis model assessment of insulin resistance (HOMA-IR), international normalized ratio (INR), hepatitis C virus antibody (anti-HCV), and hepatitis B surface antigen (HBsAg). Routine biochemical parameters were analyzed using Beckman Coulter AU680 (Beckman Coulter Inc., Brea, CA, USA) and ADVIA Centaur XPT system (Siemens Healthineers, Erlangen, Germany) automated analyzers. Complete blood counts were performed with the Mindray BC-6000 hematology analyzer (Mindray Medical International Ltd., Shenzhen, China) at the same institution.

Serum samples obtained from blood collected during routine clinical assessments were stored at −80 °C until analysis. Following the completion of participant recruitment, TMAO, TNF-α, IL-6, IL-10, MDA (malondialdehyde), and GSH (glutathione) levels were analyzed. Serum TMAO concentrations were measured using a commercially available enzyme-linked immunosorbent assay (ELISA) kit (Catalog No. SL3802Hu; SunLong Biotech Co., Ltd., Hangzhou, China). The kit’s analytical sensitivity was 0.5 pg/mL with a measurement range of 3.5–125 pg/mL. The intra-assay coefficient of variation (CV) was <10%, and the inter-assay CV was <12%. Serum IL-6 (Catalog No. L2K6P2), IL-10 (Catalog No. L2KXP2), and TNF-α (Catalog No. L2KNF2) concentrations were determined using the IMMULITE^®^ 2000 XPi analyzer (Siemens Healthineers, Erlangen, Germany). All assays were run and quality-controlled according to the respective manufacturers’ recommendations and the laboratory’s standard operating procedures.

GSH levels in serum samples were measured spectrophotometrically using the Ellman method [35]. Serum MDA levels were determined using a colorimetric procedure based on the thiobarbituric acid reactive substances (TBARS) method [36,37].

### 2.8. Statistical Analysis

All statistical analyses were performed using SPSS Statistics 29.0 (IBM Corp., Armonk, NY, USA). The normality of the data distribution was assessed using the Kolmogorov–Smirnov and Shapiro–Wilk tests. Mean ± standard deviation and median (min–max or IQR) values were reported for continuous numerical variables, and numbers and percentages were reported for categorical variables. For comparisons between groups, one-way analysis of variance (ANOVA) was used for variables showing a normal distribution, and the Kruskal–Wallis test was used for variables not showing a normal distribution. A post hoc Dunn test with Bonferroni correction was applied for multiple comparisons. Categorical variables were compared using the Chi-square test. In comparing inflammation and oxidative stress parameters between fibrosis groups, adjustment for potential confounding variables was performed. To account for the confounding effects of demographic, anthropometric, and clinical variables, multivariable models were constructed. For parametric data, analysis of covariance (ANCOVA) was used, while Quade’s rank analysis of covariance was applied for non-parametric data. All models included age, sex, BMI, presence of diabetes mellitus, hypertension, or hyperlipidemia, relevant medication use (antidiabetic, antihypertensive, and lipid-lowering agents), physical activity level (IPAQ-SF score), and Mediterranean diet adherence (MEDAS score) as covariates. Homogeneity of variance was assessed using Levene’s test before ANOVA/ANCOVA analyses. Relationships between parameters were evaluated using Spearman correlation analysis. The appropriate effect size was calculated and reported for each statistical analysis. For ANOVA analyses, eta-squared (η^2^), for Kruskal–Wallis tests, epsilon-squared (ε^2^), for Chi-square tests, Cramer’s V, and for correlation analyses, the correlation coefficient (r), were used as effect size measures. Effect sizes were interpreted according to Cohen’s classification: small effect (η^2^ = 0.01, ε^2^ = 0.01, V = 0.10, r = 0.10), medium effect (η^2^ = 0.06, ε^2^ = 0.06, V = 0.30, r = 0.30), large effect (η^2^ = 0.14, ε^2^ = 0.14, V = 0.50, r = 0.50). A value of *p* < 0.05 was considered statistically significant.

Sample size was calculated using an a priori power analysis with G*Power software (version 3.0.10, Universität Düsseldorf, Düsseldorf, Germany). For a study design comparing four groups (healthy controls, MASLD patients with early-stage fibrosis (F0–F1), moderate fibrosis (F2), and advanced fibrosis (F3–F4)), and assuming a minimum effect size of Cohen’s f = 0.30 (medium effect) for differences in Mediterranean diet adherence, TMAO, oxidative stress, and inflammatory markers, the required sample size was calculated as at least 41 participants per group, with 90% power and a significance level (α) of 0.05, resulting in a total of 164 participants.

## 3. Results

### 3.1. Demographic Characteristics

The distribution of demographic characteristics of the individuals participating in the study according to groups is shown in Table 1. According to this table, no significant difference was found between the groups in terms of gender distribution (*p* = 0.693). The mean ages of the control group, early-stage fibrosis group (F0–F1), significant fibrosis group (F2), and advanced fibrosis group (F3–F4) were 41.7 years, 51.3 years, 47.9 years, and 55.8 years, respectively. According to this result, the mean age in the control group was found to be younger than the age of patients with early- and advanced stage fibrosis. In addition to this result, it was determined that the age of individuals in the advanced stage group was greater than that of individuals in the control and significant fibrosis groups. The control group stood out as the youngest group, while the advanced fibrosis group, which had the highest fibrosis stage, was significantly older than both the control and significant fibrosis groups (*p* < 0.001; effect size = 0.153) (Table 1).

The distribution of vitamin and mineral supplementation, smoking, and alcohol use among individuals is shown in Table 1 according to groups. Accordingly, it was determined that 50.0% of the individuals in the control group were taking vitamin and mineral supplements. The incidence of vitamin and mineral use was found to be 68.1%, 68.1%, and 55.6% in individuals with early-stage, significant, and advanced fibrosis, respectively. According to this result, it was determined that the rate of vitamin and mineral use in individuals with advanced fibrosis was lower than in individuals in other patient groups, but this result was not statistically significant (*p* > 0.05). No significant difference was found between groups in the rates of smoking and alcohol use (*p* > 0.05) (Table 1).

The distribution of information regarding the health status of the individuals participating in the study according to the study group is shown in Table 2. Accordingly, the incidence of chronic disease was determined to be lower in the individuals in the control group compared to the other groups. The incidence of chronic disease was determined to be higher in the individuals in the advanced fibrosis group compared to the control and significant fibrosis group, and this result was statistically significant (*p* < 0.001; effect size = 0.760). No significant difference between the groups was determined in terms of chronic diseases other than diabetes, hypertension, and hyperlipidemia (*p* > 0.05). The incidence of hypertension (*p* < 0.001; effect size = 0.459) and hyperlipidemia (*p* < 0.001; effect size = 0.364) was found to be lower in the control group compared to the other groups, and there was no significant difference compared to the fibrosis stages. The incidence of diabetes (*p* < 0.001; effect size = 0.546) was found to be the highest in the advanced fibrosis group and the lowest in the control group. It was determined that there was no statistically significant difference between the duration of diagnosis and patient groups (*p* > 0.05).

Medication use differed markedly across the study groups. As expected, none of the participants in the control group were receiving antidiabetic, antihypertensive, or lipid-lowering medications. In contrast, the use of combination therapies increased with fibrosis severity. The most frequent pattern of medication use in the advanced fibrosis group was the combined use of antidiabetic, antihypertensive, and lipid-lowering medications (28.9%), followed by the combined use of antidiabetic and antihypertensive medications (26.7%). Patients with early fibrosis were more commonly receiving antihypertensive medications alone (23.4%), whereas the patterns of medication use in the significant-stage fibrosis group were more evenly distributed across the treatment categories. Overall, the findings indicate a progressive increase in the complexity of patterns of medication use with increasing liver fibrosis severity, reflecting the greater burden of metabolic comorbidities in patients with advanced disease (Table 3).

### 3.2. Anthropometric Indices of Study Groups

Anthropometric measurements and body composition parameters were evaluated according to gender in the fibrosis and control groups. Accordingly, in terms of body weight in male individuals, the mean body weights of the early-stage fibrosis group (F0–F1), the significant fibrosis group (F2), and the advanced stage fibrosis group (F3–F4) were significantly higher than the control group (*p* < 0.001 in all; effect size = 0.400). Body weight averages in the other groups were similar. When BMI was evaluated, the mean BMI values of the early-stage, significant-stage, and advanced stage fibrosis groups were significantly higher than the control group (*p* < 0.001 for all three; effect size = 0.477). In addition, it was observed that patients in the important fibrosis group had significantly higher BMI values than early-stage fibrosis patients (*p* = 0.018). BMI averages in other groups were similar (Table 4).

In terms of fat ratio, the mean fat ratios of the early-stage, significant, and advanced fibrosis groups were significantly higher than the control group (*p* < 0.001 for all three; effect size = 0.470). In addition, it was observed that the patients in the significant fibrosis group had a significantly higher fat content than the early-stage fibrosis patients (*p* = 0.040). The fat ratio averages in the other groups were similar.

When the neck circumference was evaluated, the mean neck circumference of the early-stage, significant, and advanced stage fibrosis groups was significantly higher than the control group (*p* < 0.001 for all three; effect size = 0.382). Although the mean neck circumference of the patients in the significant fibrosis group was higher than that of the early-stage fibrosis patients, it was not statistically significant (*p* = 0.294). The mean neck circumference in the other groups was similar. When male individuals were evaluated in terms of waist/hip ratio, the mean waist/hip ratios of the early-stage, significant, and advanced fibrosis groups were significantly higher than the control group (*p* < 0.001 for all three; effect size = 0.623). When the fibrosis groups were compared among themselves, no significant difference was observed in terms of waist/hip ratio.

In terms of body weight in female individuals, the mean body weights of the early-stage fibrosis group, the significant fibrosis group, and the advanced fibrosis group were significantly higher than the control group (*p* < 0.001 for all three; effect size = 0.493). In addition, it was observed that the patients in the advanced fibrosis group had significantly higher body weight than the patients in the early-stage fibrosis group (*p* = 0.048). Body weight averages in the other groups were similar.

When BMI was evaluated, the mean BMI values of the early-stage, significant, and advanced fibrosis groups were significantly higher than the control group (*p* < 0.001 for all three; effect size = 0.566). In addition, it was observed that the patients in the advanced stage fibrosis group had significantly higher BMI values than the patients in the early-stage fibrosis group (*p* = 0.049). The BMI averages in the other groups were similar.

In terms of fat ratio, the mean fat ratios of the early-stage, significant, and advanced fibrosis groups were significantly higher than the control group (*p* < 0.001 for all three; effect size = 0.710). The fat ratio averages in the other groups were similar.

When the neck circumference was evaluated, the mean neck circumference of the early-stage, significant, and advanced stage fibrosis groups was significantly higher than that of the control group (*p* < 0.001 for all three; effect size = 0.543). When the fibrosis groups were compared among themselves, no significant difference was observed in terms of neck circumference.

In terms of waist/hip ratio, the averages of the early-stage, significant, and advanced stage fibrosis groups were significantly higher than the control group (*p* < 0.001 for all three; effect size = 0.659). The waist/hip ratio averages in the other groups were similar.

### 3.3. Biochemical Parameters of the Study Groups

In Table 5, changes in routine biochemical parameters according to control and fibrosis groups are evaluated. When the fasting blood glucose (FBG) levels were examined, the median FBG values of the early-stage fibrosis group, the significant fibrosis group, and the advanced stage fibrosis group were significantly higher than the control group (*p* < 0.001, *p* = 0.002, and *p* < 0.001, respectively; effect size = 0.176). In addition, it was observed that the patients in the advanced fibrosis group had significantly higher FBS values than the significant fibrosis group (*p* < 0.001). The median FBS values in the other groups were similar.

When HbA1c values were evaluated, the median HbA1c values of the early-stage, significant, and advanced stage fibrosis groups were significantly higher than the control group (*p* = 0.001, *p* < 0.001, and *p* < 0.001, respectively; effect size = 0.250). In addition, it was observed that the patients in the advanced stage fibrosis group had significantly higher HbA1c values than the patients in the early-stage fibrosis group (*p* = 0.001). The median HbA1c values in the other groups were similar.

In terms of HOMA-IR and median insulin values, the median HOMA-IR values of the early-stage, significant, and advanced stage fibrosis groups were significantly higher than the control group (*p* < 0.001 for all three). In addition, it was observed that the patients in the advanced stage fibrosis group had significantly higher HOMA-IR values than the patients in the early-stage fibrosis group (*p* = 0.006). In addition, it was observed that the patients in the advanced stage fibrosis group had significantly higher insulin values than the patients in the early-stage fibrosis group (*p* = 0.026). The median HOMA-IR values and the median insulin values in the other groups were similar.

When total cholesterol concentrations were evaluated, the mean total cholesterol value of the early-stage fibrosis group was significantly higher than that of the control group (*p* = 0.001; effect size = 0.119). Interestingly, the mean total cholesterol value of the advanced fibrosis group was found to be significantly lower than that of the early-stage and significant fibrosis group (*p* < 0.001 and *p* = 0.040, respectively). No significant difference was observed between the control group and the significant and advanced fibrosis groups.

In terms of triglyceride (TG) values, the median TG values of the early-stage, significant, and advanced stage fibrosis groups were significantly higher than the control group (*p* < 0.001 for all three; effect size = 0.167). The median TG values in the other groups were similar.

When HDL cholesterol concentrations were examined, it was observed that the median HDL-C values of the early-stage, significant, and advanced stage fibrosis groups were significantly lower than the control group (*p* = 0.005, *p* = 0.002, and *p* = 0.038, respectively; effect size = 0.080). No significant differences were observed between the groups.

When LDL cholesterol mean values were evaluated, the mean LDL-C value of the early-stage fibrosis group was significantly higher than the control group (*p* = 0.035; effect size = 0.088). In addition, the mean LDL-C value of the advanced fibrosis group was found to be significantly lower than that of the early fibrosis group (*p* = 0.001). The LDL-C mean values in the other groups were similar.

In terms of ALT values, the median ALT values of the early-stage, significant, and advanced stage fibrosis groups were found to be significantly higher than the control group (*p* < 0.001 for all three; effect size = 0.207). The ALT median values in the other groups were similar.

When AST median values were examined, the median AST values of the early-stage, significant, and advanced stage fibrosis groups were significantly higher than the control group (*p* = 0.023, *p* < 0.001, and *p* < 0.001; effect size = 0.178). The median AST values of the important and advanced fibrosis groups were also significantly higher than the early-stage fibrosis group (*p* = 0.008 and *p* = 0.004, respectively). No significant difference was observed between the significant and advanced fibrosis groups.

When GGT levels were examined, the median GGT values of the early-stage, significant, and advanced stage fibrosis groups were significantly higher than the control group (*p* < 0.001 for all three; effect size = 0.196). In addition, it was observed that patients in the advanced fibrosis group had significantly higher GGT values than both early-stage fibrosis patients and MASLD patients with significant fibrosis (*p* = 0.001 and *p* = 0.008, respectively). No significant difference was observed between the early-stage and significant fibrosis groups.

In terms of median uric acid values, the median uric acid values of the early-stage, significant, and advanced stage fibrosis groups were significantly higher than the control group (*p* = 0.006, *p* = 0.001, and *p* = 0.021, respectively; effect size = 0.053). When the fibrosis groups were compared among themselves, no significant difference was observed in terms of uric acid.

In terms of INR values, the median INR value of the advanced stage fibrosis group was found to be significantly higher than both the early-stage fibrosis group and the significant fibrosis group (*p* < 0.001 and *p* = 0.002, respectively; effect size = 0.158). The median INR values in the other groups were similar.

When the ferritin concentrations in the groups were examined, the median ferritin values of the early-stage, important, and advanced stage fibrosis groups were significantly higher than the control group (*p* = 0.018, *p* < 0.001, and *p* = 0.002, respectively; effect size = 0.097). When fibrosis groups were compared among themselves, no significant difference was observed in terms of ferritin.

When the median CRP values were evaluated, the median CRP values of the early-stage, significant, and advanced stage fibrosis groups were significantly higher than the control group (*p* = 0.001, *p* < 0.001, and *p* < 0.001, respectively; effect size = 0.162). In addition, it was observed that the patients in the advanced stage fibrosis group had significantly higher CRP values than the patients in the early-stage fibrosis group (*p* = 0.038). The median CRP values in the other groups were similar.

In terms of CAP values, the mean CAP values of the early-stage, significant, and advanced stage fibrosis groups were significantly higher than the control group (*p* < 0.001 for all three; effect size = 0.653). The mean CAP values in the other groups were similar (Table 5).

### 3.4. Inflammatory, Oxidative Stress, and TMAO Parameters of the Study Groups

Table 6 presents the distribution of inflammatory and oxidative stress markers across study groups. TNF-α levels were consistently elevated across all fibrosis groups compared to controls (*p* < 0.001). Within the fibrosis spectrum, advanced stage fibrosis demonstrated the highest TNF-α concentrations, significantly higher than early-stage (*p* < 0.001) and significant fibrosis (*p* = 0.016). Following multivariable adjustment for age, sex, BMI, comorbidities, physical activity, Mediterranean diet adherence, and medication use, the difference between advanced and early-stage fibrosis remained statistically significant (*p* = 0.030), while other pairwise comparisons lost significance.

IL-6 exhibited a similar pattern, with advanced fibrosis showing the highest concentrations compared to controls (*p* < 0.001), early-stage (*p* < 0.001), and significant fibrosis (*p* = 0.011). After covariate adjustment, the association with controls was attenuated (*p* = 1.000), whereas differences versus early-stage (*p* = 0.004) and significant fibrosis (*p* = 0.049) persisted. IL-6 levels were comparable among the remaining groups.

IL-10 concentrations showed a progressive increase with fibrosis severity, reaching statistical significance only in the advanced fibrosis group versus the control group (*p* = 0.010). However, this association did not withstand multivariable adjustment, suggesting confounding by metabolic and lifestyle factors.

GSH, a key antioxidant, displayed elevated concentrations in all fibrosis groups compared to controls, with early-stage fibrosis showing the most pronounced increase (*p* = 0.001). Significant and advanced fibrosis groups exhibited numerically higher GSH levels that did not reach statistical significance (*p* = 0.376 and *p* = 0.805, respectively). Following adjustment for confounders, all between-group differences in GSH were diminished (*p* > 0.05).

MDA, a lipid peroxidation marker, peaked in early-stage fibrosis, significantly exceeding both controls (*p* = 0.001) and significant fibrosis (*p* < 0.001). After adjustment for confounders, only the difference versus significant fibrosis remained significant (*p* = 0.011). The MDA/GSH ratio, reflecting oxidative balance, showed no significant variation across groups in either unadjusted or adjusted analyses (*p* = 0.877).

In unadjusted analyses, TMAO concentrations were paradoxically lower in early-stage (*p* = 0.001) and advanced fibrosis (*p* < 0.001) compared to controls. However, after controlling for demographic, anthropometric, clinical, dietary, and medication-related confounders, these differences were no longer statistically significant (early-stage fibrosis: *p* = 0.210; significant fibrosis: *p* = 1.000; advanced fibrosis: *p* = 0.387).

### 3.5. Compliance of the Study Groups with the Mediterranean Diet

**Figure 1:**
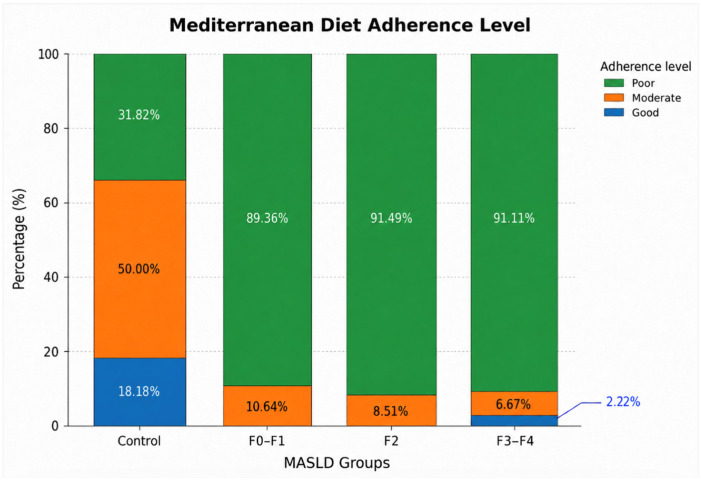
Distribution of individuals’ levels of adherence to the Mediterranean diet by groups.

Table 7 shows the distribution of individuals’ adherence levels to the Mediterranean diet according to fibrosis groups. While there were no individuals with good MD adherence among individuals with early and significant fibrosis, it was found that 89.4% and 91.5% of the individuals in this group had poor MD adherence, respectively. It was determined that 91.1% of the individuals with advanced fibrosis had poor MD adherence, and 6.7% had moderate MD adherence. According to this result, it was determined that the rate of poor MD adherence was lower in the control group than in individuals diagnosed with fibrosis (*p* < 0.001; effect size = 0.428) (Figure 1).

The MEDAS score median value of the individuals in the control, early-stage, significant, and advanced stage groups was found to be 7.5, 5.0, 4.0, and 4.0, respectively (*p* < 0.001; effect size = 0.442). According to this result, the median MEDAS score of the individuals in the control group was found to be significantly higher than that of the early-stage fibrosis (*p* < 0.001), significant fibrosis (*p* < 0.001), and advanced stage fibrosis (*p* < 0.001) groups. In addition, it was found that the median MEDAS score of the individuals in the early-stage fibrosis group was higher than that of the significant fibrosis (*p* = 0.006) and advanced stage fibrosis (*p* = 0.012) groups.

When individual MEDAS items were evaluated according to fibrosis groups, significant differences were observed among the groups in several dietary components (Table 7). The proportion of individuals using olive oil as the main culinary fat was 72.7% in the control group, 59.6% in the F0–F1 group, 36.2% in the F2 group, and 33.3% in the F3–F4 group (*p* < 0.001). In particular, the rates of using olive oil as the main fat were significantly lower in the significant and advanced fibrosis groups compared with the control group (*p* < 0.001 for both).

In post hoc comparisons, the proportion of individuals consuming ≥4 tablespoons of olive oil per day was found to be significantly lower in all MASLD fibrosis groups compared with the control group (*p* = 0.006, *p* = 0.006, and *p* < 0.001, respectively).

The proportion of individuals consuming ≥2 portions of vegetables per day was significantly lower in all MASLD fibrosis groups compared with the control group (*p* < 0.001 for all).

A significant difference was also observed among the groups in terms of fruit consumption ≥3 portions/day (*p* = 0.004); fruit consumption was significantly lower in the advanced fibrosis group compared with the control group (*p* < 0.001).

When dietary components related to TMAO were evaluated, the proportion of individuals consuming <1 portion/day of red/processed meat did not differ significantly among the groups (*p* = 0.958). The proportion of individuals consuming fish/seafood ≥ 3 portions/week was 15.9% in the control group, 10.6% in the early-stage fibrosis group, 0% in the significant fibrosis group, and 6.7% in the advanced fibrosis group (*p* = 0.036). In the post hoc analysis, this proportion was significantly lower in the significant fibrosis group compared with the control group (*p* = 0.024).

The proportion of individuals consuming <1 portion/day of butter/margarine differed significantly among the groups (*p* = 0.005); this proportion was significantly lower in the early-stage fibrosis and advanced fibrosis groups compared with the control group (*p* = 0.012 and *p* = 0.042, respectively).

The proportion of individuals consuming <1 portion/day of sugary beverages also differed among the groups (*p* = 0.010) and was significantly lower in the significant fibrosis group compared with the control group (*p* = 0.006).

The proportion of individuals consuming legumes ≥3 portions/week was significantly lower in the significant and advanced fibrosis groups compared with the control group (*p* = 0.012 and *p* < 0.001, respectively). The proportion of individuals consuming pastry products <3 times/week was significantly lower in the significant fibrosis group compared with the control group (*p* = 0.030).

Nuts consumption ≥3 portions/week differed markedly among the groups (*p* < 0.001). The early-stage fibrosis group had a significantly lower proportion compared with the control group (*p* < 0.001), whereas the significant and advanced fibrosis groups had significantly lower proportions compared with both the control group (*p* < 0.001 for both) and the early-stage fibrosis group (*p* = 0.042 and *p* = 0.006, respectively).

The proportion of individuals consuming sofrito ≥2 times/week was also significantly lower in the early-stage, significant, and advanced fibrosis groups compared with the control group (*p* = 0.018, *p* < 0.001, and *p* < 0.001, respectively). In contrast, no significant differences were observed among the groups in terms of wine consumption or preference for poultry instead of red meat (*p* > 0.05) (Table 7).

### 3.6. Physical Activity Levels of Study Groups

Table 8 shows the distribution of physical activity levels of individuals by groups and the average of MET values. According to this table, the rate of inactive (<600 MET) individuals was 43.2% in the control group, 76.6% in the early-stage fibrosis group, 74.5% in the significant fibrosis group, and 75.6% in the advanced stage fibrosis group. It was determined that the proportion of individuals who did not do physical activity was lower in the control group than in individuals with early, significant, and advanced stage fibrosis. In addition to this result, it was determined that the proportion of minimally active individuals was lower in individuals with advanced fibrosis than in the control group, and this result was statistically significant (*p* = 0.004; effect size = 0.229).

When the IPAQ-SF total scores were evaluated, it was determined that the median MET value of the control group was 840.0. This value was found to be 270.0, 225.0, and 90.0 in individuals with early, significant, and advanced stage fibrosis, respectively. According to this result, it was determined that the median MET value of the individuals in the control group was higher than the individuals in the early (*p* = 0.007), important (*p* =0.003), and advanced fibrosis group (*p* < 0.001) (*p* < 0.001; effect size = 0.117).

### 3.7. Correlation Analysis Among Liver Steatosis, Liver Fibrosis, TMAO, Mediterranean Diet Adherence, Inflammatory Biomarkers, and Oxidative Stress Markers

As a result of the Spearman correlation analysis, a significant positive correlation was found between CAP dB/m fat accumulation level and E kPa fibrosis level (r = 0.421, *p* < 0.001). CAP dB/m fat accumulation level was moderately negatively correlated with the MEDAS total score (r = −0.540, *p* < 0.001). Similarly, a significant and moderate negative correlation was found between E kPa fibrosis level and MEDAS total score (r = −0.567, *p* < 0.001). In addition, E kPa fibrosis level was found to be positively and significantly correlated with TNF-α (r = 0.547, *p* < 0.001) and IL-6 (r = 0.439, *p* < 0.001) (Table 9).

In correlation analysis, a moderate, positive, and statistically significant relationship was found between the CAP dB/m fat accumulation level and the E kPa fibrosis level. (r = 0.421, *p* < 0.001.) CAP dB/m fat accumulation level was found to be positively and significantly correlated with TNF-α (r = 0.343, *p* < 0.001), IL-6 (r = 0.183, *p* = 0.017), GSH (r = 0.202, *p* = 0.006), and MDA (r = 0.153, *p* = 0.038). On the other hand, CAP dB/m fat accumulation level showed significant negative correlations with TMAO (r = −0.249, *p* < 0.001), MEDAS total score (r =−0.540, *p* < 0.001), and IPAQ-SF total score (r = −0.170, *p* = 0.022).

E kPa fibrosis level was found to be positively and significantly correlated with TNF-α (r = 0.547, *p* < 0.001), IL-10 (r = 0.298, *p* < 0.001), and IL-6 (r = 0.439, *p* < 0.001). Negatively significant correlations were found between E kPa fibrosis level and TMAO (r = −0.278, *p* < 0.001), MEDAS total score (r = −0.567, *p* < 0.001), and IPAQ-SF total score (r = −0.261, *p* < 0.001). The strongest negative relationship was found between E kPa fibrosis level and MEDAS total score.

TNF-α level was found to be positively correlated with CAP dB/m (hepatic steatosis), liver fibrosis (E kPa), IL-10 and IL-6, and negatively significantly correlated with TMAO, MEDAS total score, and IPAQ-SF total score. IL-10 level showed a significant positive correlation with E kPa fibrosis, TNF-α, IL-6, and GSH.

MEDAS total score was found to be negatively significantly correlated with CAP dB/m fat accumulation, E kPa fibrosis, TNF-α, IL-6, and GSH, while MDA/GSH ratio was positively correlated with TMAO and IPAQ-SF total score. IPAQ-SF total score was found to be negatively correlated with CAP dB/m fat accumulation, E kPa fibrosis, TNF-α, and MDA, and positively and significantly correlated with TMAO and MEDAS total score. The findings generally show that adherence to the Mediterranean diet and the level of physical activity may be associated with fatty liver, fibrosis, and some inflammatory/oxidative stress indicators.

In addition, a significant negative correlation was found between MDA level and the IPAQ-SF total score (r = −0.231, *p* = 0.002).

## 4. Discussion

The present study comprehensively evaluated Mediterranean diet adherence, TMAO concentrations, inflammatory biomarkers, oxidative stress markers, and physical activity across objectively defined liver fibrosis stages using FibroScan. The main findings demonstrated that, after adjustment for potential confounding factors, TNF-α levels were significantly higher in patients with advanced fibrosis than in those with early-stage fibrosis. Similarly, IL-6 levels were highest in the advanced fibrosis group and were significantly higher than those in the early-stage and significant fibrosis groups. In contrast, no significant differences were observed in IL-10, GSH, or the MDA/GSH ratio among the fibrosis groups. Although TMAO concentrations were significantly lower in all fibrosis groups compared to the control group in the unadjusted analyses, these differences were no longer significant after adjustment for potential confounding factors. Furthermore, lower Mediterranean diet adherence and physical activity levels were observed in the fibrosis groups compared with the control group. Correlation analyses demonstrated inverse associations of liver steatosis and fibrosis with Mediterranean diet adherence and physical activity, whereas positive associations were observed with inflammatory biomarkers.

MASLD is one of the common chronic liver diseases globally and is closely associated with mechanisms such as visceral adiposity, insulin resistance, inflammation, oxidative stress, dyslipidemia, changes in the liver-gut axis, along with hepatic symptoms [38]. However, studies that holistically address the relationship between Mediterranean diet adherence, TMAO levels, inflammatory and oxidative stress parameters, and fibrosis severity are limited.

When the study groups were evaluated, it was determined that parameters such as body weight, height, BMI, fat percentage, fat mass, waist circumference, hip circumference, waist/hip ratio, neck circumference, and visceral fat ratio were higher in fibrosis groups compared to the control group. When the results of another study conducted with the cross-sectional MASLD population were examined, similar to our study, in addition to the high prevalence of hypertension and diabetes in individuals diagnosed with MASLD, anthropometric measurements, body weight, and BMI evaluations were found to be significantly higher in the MASLD group compared to the non-MASLD group [39]. In another study, it was observed that mean body fat values were consistently higher in women and were associated with the risk of MASLD. Visceral fat and MASLD risk scales showed similar results, and it was stated that men had higher values [40]. In the study of Ibarra-Reynoso et al., anthropometric measurements were also significantly elevated in the MASLD cohort, including weight, BMI, waist circumference, and hip circumference. In another similar study based on FibroScan, BMI, and waist circumference were found to be associated with an increased risk of progression to moderate to advanced liver fibrosis in the general population [41].

According to the 2024 EASL-EASD-EASO Clinical Practice Guidelines, the risk assessment for fibrosis in MASLD should be performed in people with cardiometabolic risk factors, abnormal liver enzymes, and/or radiological findings of hepatic steatosis [16]. In our study, differences were observed in many biochemical parameters related to glucose metabolism, lipid profile and liver function tests in MASLD groups. These findings support that MASLD is not limited to hepatic steatosis and fibrosis, but is a spectrum of metabolic disease associated with insulin resistance, dyslipidemia, hepatocellular damage, and systemic inflammation [42]. As a matter of fact, in parallel with our study, previous studies reported that fasting glucose, fasting insulin, HOMA-IR, ALT, AST, GGT, and uric acid levels were higher and HDL-C levels were LOWER in MASLD patients compared to healthy controls [43,44]. Similarly, cohort studies related to MASLD have shown that as disease severity increases, there may be an increase in markers associated with inflammation and metabolic stress, such as CRP, ferritin, and uric acid. [45,46].

Although dyslipidemia in MASLD is generally defined as increased Total cholesterol, triglyceride, and LDL-C and low HDL-C, in our study, total cholesterol and LDL-C were found to be lower in fibrosis groups compared to the control group, while triglyceride levels were found to be higher [47]. In a study with similar results, it was stated that the use of lipid-lowering drugs may mask conventional lipid parameters in MASLD patients [48]. Lipoprotein abnormalities are common in chronic liver disease, and in a similar study, lower LDL-C and triglyceride levels were associated with advanced liver fibrosis, even after adjustment for statin and fibrate use. This was interpreted as probably due to a decrease in hepatic lipoprotein production as liver disease progressed [49]. In our study, the combined use of antidiabetic (metformin, insulin, GLP-1 agonists, etc.), antihypertensive (ACE inhibitors, ARBs, beta-blockers, calcium channel blockers, etc.), and lipid-lowering medications (statins, fibrates, etc.) was also found to be the most frequent medication use pattern in the advanced fibrosis group. The lower LDL-cholesterol levels observed in the advanced fibrosis group may be attributed not only to impaired hepatic lipid metabolism associated with advanced liver disease but also to dietary modifications adopted following diagnosis and the more frequent use of lipid-lowering medications [3,50]. Although TMAO concentrations also tended to be lower in the advanced fibrosis group, this difference was no longer statistically significant after adjustment for potential confounding factors. Therefore, the coexistence of lower LDL-cholesterol and TMAO levels in advanced fibrosis should be interpreted with caution, as it may reflect the combined effects of disease-related metabolic alterations, lifestyle modifications, medication use (particularly statins), and other potential confounding factors rather than a direct biological association [51,52,53].

Increased fat in the liver is responsible for increased insulin resistance, which plays an important role in the prognosis of MASLD. Adipocytes and tumor necrosis factor (TNF-α), which act as endocrine organs, produce various cytokines, including angiotensinogen, free fatty acids, and leptin, which are mainly responsible for lipotoxicity. This pro-inflammatory effect may play an important role in the progression to liver fibrosis, which is the most important risk factor for the development of liver cirrhosis and liver cancer in patients with MASLD [40,54]. In our study, after controlling for confounders, TNF-α levels, a pro-inflammatory cytokine, were found to be significantly higher in MASLD patients with advanced stage fibrosis compared with those with early-stage fibrosis. IL-6, a pro-inflammatory cytokine, was found to be significantly higher in the advanced fibrosis group compared to the early and significant fibrosis groups. Although IL-10 levels appeared to be increased in the advanced fibrosis group compared with the control group, this difference did not reach statistical significance. When the studies in the literature were examined, one study reported that MASLD patients had a more negative metabolic and inflammatory profile compared to the control group. While atherogenic lipid levels, IL-6, and IL-10 concentrations were found to be higher in these patients, TNF-α levels were shown to be similar between the groups [42]. Similarly, another study found that IL-6, TNF-α, and CRP levels, which are inflammatory markers, were significantly higher in MASLD patients [55]. The increase in IL-6 and TNF-α levels, which are pro-inflammatory cytokines produced by Kupffer cells and hepatocytes in the liver and play an important role in hepatic inflammation, reflects ongoing liver damage and inflammation [54,56,57]. In contrast, although the increase in IL-10 levels was not statistically significant, this elevation may be considered a compensatory mechanism aimed at balancing the body’s excessive inflammatory response, given the anti-inflammatory properties of IL-10. IL-10 is thought to have anti-inflammatory and anti-fibrotic properties on the liver, and while significantly higher results were found in the patient group in some studies, no significant difference was found in some studies, and the data on its role in MASLD remained limited and uncertain [58,59,60].

In this study, GSH levels and the MDA/GSH ratio were the same among the groups, whereas MDA levels were found to be significantly higher in the early-stage fibrosis group compared with the significant fibrosis group. In some studies, it was observed that MDA level increased as lipid peroxidation and oxidative damage increased as fibrosis levels progressed, and GSH level decreased as antioxidant reserve was depleted [61,62]. In general, in order to protect organisms against the increased production of reactive oxygen species (ROS) at the onset of MASLD, the expression or activity of antioxidant enzymes usually increases (GSH), while in the advanced stage of the disease, the expression/activity of various antioxidants usually decreases due to oxidative damage in the liver and non-liver cells, making liver damage worse [63]. The decrease we observed in MDA in the significant fibrosis group compared to the early-stage should not be interpreted as a reversal of oxidative damage. This is consistent with the condition called “burnt-out NASH-MASLD” in the literature, in which hepatic fat content decreases in some patients during the MASLD/NAFLD advanced fibrosis period [64,65]. This suggested that steatosis and lipid peroxidation due to lipotoxicity may be more prominent in early-stage fibrosis, and MDA levels may increase, while decreased hepatic fat/substrate in advanced fibrosis may lead to lower MDA levels. Similarly, some studies of advanced liver disease have reported that MDA levels do not increase linearly with disease severity; numerically lower values may be observed in the advanced group [66].

Trimethylamine N-oxide (TMAO) is a gut microbiota-derived metabolite produced through the metabolism of dietary precursors such as choline, L-carnitine, and betaine. These nutrients, mainly found in red meat, eggs, dairy products, and certain fish, are first converted by intestinal microbiota into trimethylamine (TMA), which is subsequently absorbed and oxidized to TMAO in the liver by flavin-containing monooxygenase 3 (FMO3) [67,68]. Therefore, circulating TMAO levels are influenced by dietary intake, gut microbiota composition, hepatic enzymatic activity, and renal excretion [19,68,69]. A meta-analysis study on TMAO found that MASLD patients had higher circulating TMAO levels compared to a control group of patients [22]. Without adjusting for multiple covariates, TMAO levels were significantly higher in the control group compared to advanced fibrosis; however, after adjustment, this difference was no longer statistically significant. In a similar study, in line with our findings, it was stated that impaired betaine homocysteine methyltransferase activity in cirrhotic liver tissue may lead to impaired FMO3 expression and consequently impaired trimethylamine → TMAO formation [70]. In an intervention study, a significant positive correlation was found between the decrease in TMAO levels and adherence to a plant-based diet. Studies have shown that TMAO plasma levels cannot be explained by a single factor, but may be affected by multiple factors, including dietary factors (fish consumption, eggs, dairy products, whole grain consumption, etc.), microbiota (dysbiosis), or cooking methods [53,71,72,73,74]. The paradoxically lower TMAO levels in advanced fibrosis, despite similar red meat (a major source of carnitine) consumption across all our study groups, suggest that impaired hepatic FMO3 activity may be the primary determinant of TMAO levels in advanced MASLD. Additionally, significantly lower fish consumption in advanced fibrosis groups may contribute to lower TMAO levels, as fish is a TMAO-related dietary component. In addition, studies have shown that commonly used medications, particularly polypharmacy, may affect the composition of the gut microbiota [75]. Antidiabetic and lipid-lowering medications, especially metformin and statins, may influence gut microbiota composition and TMA/TMAO metabolism [76]. Metformin, in particular, has been reported to reshape the gut microbiota, modulate microbial processes involved in the conversion of choline to TMA, and thereby potentially suppress serum TMAO levels [77,78]. Therefore, the combined use of antidiabetic, antihypertensive, and lipid-lowering medications may represent an important confounding factor when interpreting circulating TMAO levels in MASLD patients [51]. In our study, antidiabetic, antihypertensive, and lipid-lowering medications were used more frequently in the fibrosis groups, both individually and in combination. These suggest that the combination of reduced hepatic FMO3 activity, lower fish intake, and medication use may provide a comprehensive explanation for our findings.

In MASLD patients, high adherence to the Mediterranean diet has been linked to a lower risk of significant liver fibrosis [12]. In cross-sectional observational studies on hepatic steatosis, MASLD, and Mediterranean diet compliance, low MEDAS compliance was generally associated with high hepatic steatosis, fibrosis risk, and MASLD severity [12,79,80]. In parallel with our study, MD in MAFLD patients was evaluated in relation to low adherence, a pro-inflammatory profile, and the degree of hepatic fibrosis [81]. In addition, in a study conducted by Mokhtare et al., a negative correlation was found between MD compliance score, fibrosis, and steatosis [82]. Similarly, in a study investigating the relationship between MD adherence and the prevalence of steatotic liver disease, MASLD, and alcohol-related liver diseases, it was emphasized that MD adherence significantly reduces the risk of disease, has the potential to prevent metabolic liver diseases and complications, and has a protective effect [83]. All these results are in line with the fact that adherence to the MD diet was significantly higher in the individuals in the control group compared to the individuals in the fibrosis group in our study.

Our study showed that the number of inactive (sedentary) and minimally active individuals was significantly higher in the group with liver fibrosis compared to the control group. In addition, a significant negative correlation was found between the IPAQ-SF score and fibrosis. Similarly, in the literature, low daily energy expenditure in MASLD patients suggests a link between sedentary life and increased liver fibrosis and/or fatty liver [84,85]. Reducing sedentary behavior, adopting a healthy dietary pattern, and engaging in regular physical activity have been emphasized as key components of a non-pharmacological approach to reducing liver fibrosis severity and managing the risks associated with more advanced stages of fibrosis. [84,86].

In our study, multivariable covariate adjustment analyses were performed for TNF-α, IL-6, IL-10, MDA, GSH, the MDA/GSH ratio, and TMAO. Some results that were significant in the unadjusted analyses lost their statistical significance after adjustment for relevant covariates. This indicates that inflammatory markers, oxidative stress markers, and TMAO in MASLD may be substantially influenced by potential confounding factors. Therefore, the evaluation of these findings using multivariable adjustment analyses can be considered one of the strengths of our study.

This study has several limitations. First, its cross-sectional design limits causal inference regarding the relationships between Mediterranean diet adherence, TMAO concentrations, inflammatory biomarkers, oxidative stress markers, and liver fibrosis severity. Second, the single-center design may limit the generalizability of the findings. Third, although circulating TMAO concentrations were measured, gut microbiota composition and function were not directly assessed. In addition, dietary assessment was based on MEDAS, which is validated for evaluating adherence to the Mediterranean diet but does not provide detailed information on the intake of TMAO precursors, including choline, carnitine, and betaine, or TMAO-rich foods. Therefore, future studies incorporating gut microbiota profiling together with comprehensive food frequency questionnaires or dietary recalls are needed to better characterize the interactions between specific dietary components, gut microbiota, TMAO, and liver fibrosis in MASLD populations. Finally, prospective longitudinal and multicenter studies are warranted to validate our findings and further clarify these associations.

## 5. Conclusions

This study evaluated Mediterranean diet adherence, TMAO levels, inflammatory markers, oxidative stress, and physical activity levels according to fibrosis stage in MASLD patients. Alterations in TMAO, inflammatory, and oxidative stress markers have been observed in MASLD patients according to the stage of fibrosis. In particular, the increase in TNF-α and IL-6 levels suggests that the inflammatory load may be increased in relation to the severity of fibrosis. Lower TMAO levels in the fibrosis group may be attributed to the combination of reduced hepatic FMO3 activity, lower fish intake, and medication use. Elevated MDA levels in early-stage fibrosis suggest that lipid peroxidation may play a more prominent role in the initial phases of liver disease progression rather than in advanced fibrosis.

Mediterranean diet adherence and physical activity were inversely associated with pro-inflammatory markers and MASLD severity, with the highest levels observed in the control group, pointing to a potential protective role for these lifestyle factors in MASLD.

## Figures and Tables

**Table 1:** Distribution of demographic characteristics of individuals according to groups.

	Control		F0–F1		F2		F3–F4		χ^2^	*p* *	Effect Size
	(n = 44)		(n = 47)		(n = 47)		(n = 45)				
	Number	%	Number	%	Number	%	Number	%			
Gender											
Man	21	47.7	23	48.9	20	42.6	17	37.8	1.453	0.693	0.089
Woman	23	52.3	24	51.1	27	57.4	28	62.2			
Total	44	100	47	100	47	100	45	100			
Age (year)											
19–49	39	88.6	18	38.3 ^a^	21	44.7 ^a^	8	17.8 ^ac^	47.579	<0.001	0.510
50 and above	5	11.4	29	61.7 ^a^	26	55.3 ^a^	37	82.2 ^ac^			
$\bar{x}\;$± SD (year)	41.7 ± 10.17		51.3 ± 10.41 ^a^		47.9 ± 15.47		55.8 ± 11.39 ^ac^		10.773	<0.001	0.153
Vitamin and mineral use status									4.758	0.190	0.161
Yes	22	50	32	68.1	32	68.1	25	55.6			
No	22	50	15	31.9	15	31.9	20	44.4			
Smoking status									10.743	0.097	0.171
Yes	17	38.6	10	21.3	14	29.8	8	17.8			
No	24	54.5	35	74.5	28	59.6	29	64.4			
Used to	3	6.8	2	4.3	5	10.6	8	17.8			
Period of smoking										0.300	0.077
$\bar{x}\;$± SD (year)	16.1 ± 10.92		24.4 ± 10.81		20.6 ± 15.47		23.8 ± 9.79				
Cigarette smoked (number/day)										0.499	0.048
Median (min–max)	10 (2–20)		15 (3–80)		20 (4–30)		20 (2–40)				
Alcohol use status									3.119	0.374	0.131
Yes	3	6.8	1	2.1	4	8.5	1	2.2			
No	41	93.2	46	97.9	43	91.5	44	97.8			
Hepatitis Status (HBsAg/Anti-HCV)											
Positive	0	0	0	0	0	0	0	0			
Negative	44	100	47	100	47	100	45	100			

*: Chi-square test, One Way ANOVA test or Kr: Kruskal–Wallis test, F0: 25, F1: 22, F2: 47, F3: 19, F4: 26, C: 44, a: significant difference compared to control (C) group (*p* < 0.05), c: significant difference compared to F2 group (*p* < 0.05).

**Table 2:** Distribution of information on individuals’ health status according to groups.

	Control (n = 44)		F0–F1 (n = 47)		F2 (n = 47)		F3–F4 (n = 45)		χ^2^	*p* *	Effect Size
	Number	%	Number	%	Number	%	Number	%			
Comorbidity status											
Yes	-	-	39	83.0 ^a^	34	72.3 ^ab^	43	95.6 ^ac^	105.639	<0.001	0.690
No	44	100.0	8	17.0	13	27.7	2	4.4			
Comorbidity											
Ulcer-Gastritis	-	-	7	17.9	7	20.6	7	16.3	7.522	0.057	0.203
Diabetes Mellitus	-	-	16	41.0 ^a^	21	61.8 ^a^	34	79.1 ^a^	54.631	<0.001	0.546
Hypertension	-	-	23	59.0 ^a^	23	67.6 ^a^	26	60.5 ^a^	38.572	<0.001	0.459
Anemia	-	-	2	5.1	-	-	-	-	5.881	0.119	0.179
Hyperlipidemia	-	-	18	46.2 ^a^	15	44.1 ^a^	19	44.2 ^a^	24.223	<0.001	0.364
Kidney diseases	-	-	1	2.6	3	8.8	3	7.0	3.942	0.268	0.147
Liver and gallbladder disease	-	-	4	10.3	1	2.9	4	9.3	5.873	0.118	0.179
Food allergy	-	-	2	5.1	2	5.9	-	-	3.872	0.276	0.145
Psychiatric illnesses	-	-	4	10.3	6	17.6	2	4.7	6.665	0.083	0.191
Cardiorespiratory disease	-	-	2	5.1	5	14.7	6	14.0	7.479	0.058	0.202
Endocrine and metabolic diseases	-	-	2	5.1	4	11.8	3	7.0	3.912	0.271	0.146
MASLD diagnosis time (months)											
0–12	-	-	24	51.1	21	44.7	15	33.3	4.740	0.315	0.131
13–59	-	-	14	29.8	16	34.0	14	31.1			
≥60	-	-	9	19.1	10	21.3	16	35.6			

*: Chi-square test, F0: 25, F1: 22, F2: 47, F3: 19, F4: 26, C: 44; a: significant difference compared to the control group (*p* < 0.05), b: significant difference compared to the F0–F1 group (*p* < 0.05), c: significant difference compared to the F2 group (*p* < 0.05).

**Table 3:** Distribution of medication use according to the study groups.

	Control (n = 44)		F0–F1 (n = 47)		F2 (n = 47)		F3–F4 (n = 45)	
	n	%	n	%	n	%	n	%
No medications	44	100	18	38.3	17	36.2	4	8.9
Antidiabetic medications	0	0	1	2.1	6	12.8	5	11.1
Antidiabetic + Antihypertensive medications	0	0	6	12.8	6	12.8	12	26.7
Antidiabetic +Antihypertensive + Lipid-lowering medications	0	0	3	6.4	5	10.6	13	28.9
Antidiabetic + Lipid-lowering medications	0	0	4	8.5	3	6.4	3	6.7
Antihypertensive medications	0	0	11	23.4	8	17.0	5	11.1
Antihypertensive + Lipid-lowering medications	0	0	2	4.3	1	2.1	3	6.7
Lipid-lowering medications.	0	0	2	4.3	1	2.1	0	0

Lipid-lowering medications: statins, fibrates, ezetimibe. Antihypertensive: ACE inhibitors, ARBs, beta-blockers, calcium channel blockers, diuretics. Antidiabetic medications (metformin, insulin, GLP-1 agonists, SGLT2 inhibitors.

**Table 4:** Mean $\left(\bar{x}\right),\;$standard deviation (S), and median values of anthropometric measurements by groups.

	Control	F0–F1	F2	F3–F4	*p* *	Effect Size
	(n = 44)	(n = 47)	(n = 47)	(n = 45)		
	$\bar{x}\;$± SD	$\bar{x}\;$± SD	$\bar{x}\;$± SD	$\bar{x}\;$± SD		
Male						
Body weight (kg)	75.8 ± 9.90	93.7 ± 14.51 ^a^	104.3 ± 14.57 ^a^	101.4 ± 16.4 ^a^	<0.001	0.400
BMI (kg/m^2^)	25.4 ± 2.94	31.0 ± 3.69 ^a^	35.3 ± 5.01 ^ab^	34.8 ± 5.1 ^a^	<0.001	0.477
Fat content (%)	20.2 ± 3.59	27.6 ± 4.24 ^a^	32.7 ± 6.94 ^ab^	31.3 ± 6.16 ^a^	<0.001	0.470
Fat Mass (kg)	15.5 ± 4.13	26.2 ± 7.27 ^a^	34.7 ± 10.65 ^ab^	34.4 ± 15.49 ^a^	<0.001	0.396
Muscle mass (kg)	57.4 ± 6.08	63.4 ± 8.72	66.1 ± 7.54 ^a^	65.6 ± 8.41 ^a^	0.004	0.173
Neck circumference (cm)	38.5 ± 2.34	42.4 ± 2.76 ^a^	44.1 ± 2.76 ^a^	43.4 ± 3.41 ^a^	<0.001	0.382
Waist circumference (cm)	89.2 ± 6.65	107.8 ± 8.95 ^a^	117.2 ± 9.25 ^ab^	118.1 ± 12.32 ^ab^	<0.001	0.618
Hip circumference (cm)	101.7 ± 5.39	108.4 ± 6.24 ^a^	115.1 ± 8.40 ^ab^	113.2 ± 8.15 ^a^	<0.001	0.362
Waist/hip ratio	0.88 ± 0.04	0.99 ± 0.05^a^	1.02 ± 0.04 ^a^	1.04 ± 0.07 ^a^	<0.001	0.623
Visceral fat rating	8.0 ± 2.52	13.3 ± 3.84 ^a^	17.0 ± 5.37 ^a^	17.2 ± 4.07 ^ab^	<0.001	0.465
Female						
Body weight (kg)	57.3 ± 6.98	84.5 ± 11.11 ^a^	89.7 ± 18.67 ^a^	95.8 ± 17.84 ^ab^	<0.001	0.493
BMI (kg/m^2^)	22.5 ± 2.37	34.8 ± 4.13 ^a^	36.3 ± 6.64 ^a^	39.2 ± 7.08 ^ab^	<0.001	0.566
Fat content (%)	24.4 ± 5.83	41.3 ± 3.59 ^a^	41.9 ± 5.14 ^a^	44.1 ± 5.08 ^a^	<0.001	0.710
Fat Mass (kg)	14.3 ± 5.19	35.2 ± 7.45 ^a^	38.5 ± 12.13 ^a^	43.0 ± 12.50 ^ab^	<0.001	0.542
Muscle mass (kg)	40.8 ± 2.31	46.8 ± 4.24 ^a^	48.7 ± 6.66 ^a^	50.3 ± 6.17 ^a^	<0.001	0.319
Neck circumference (cm)	31.5 ± 1.86	37.1 ± 2.24 ^a^	37.9 ± 2.80 ^a^	38.4 ± 2.94 ^a^	<0.001	0.543
Waist circumference (cm)	72.7 ± 6.26	106.5 ± 8.27 ^a^	112.0 ± 12.65 ^a^	118.3 ± 12.17 ^ab^	<0.001	0.740
Hip circumference (cm)	96.6 ± 6.34	114.1 ± 9.63 ^a^	115.9 ± 12.51 ^a^	122.3 ± 14.12 ^a^	<0.001	0.416
Waist/hip ratio	0.75 ± 0.05	0.93 ± 0.06 ^a^	0.97 ± 0.07 ^a^	0.97 ± 0.07 ^a^	<0.001	0.659
Visceral fat rating	3.2 ± 1.61	11.3 ± 2.09 ^a^	11.3 ± 3.74 ^a^	13.5 ± 4.05 ^a^	<0.001	0.609

*: One Way ANOVA test. F0: 25, F1: 22, F2: 47, F3: 19, F4: 26, C: 44; a: significant difference compared to the control group (*p* < 0.05), b: significant difference compared to the F0–F1 group (*p* < 0.05).

**Table 5:** Mean ($\bar{x}$), standard deviation (S), and median values of biochemical parameters by groups.

	Control	F0–F1	F2	F3–F4	*p*	Effect Size
	(n = 44)	(n = 47)	(n = 47)	(n = 45)		
	$\bar{x}\;$± SD;	$\bar{x}\;$± SD;	$\bar{x}\;$± SD;	$\bar{x}\;$± SD;		
	Median (IQR)	Median (IQR)	Median (IQR)	Median (IQR)		
Biochemical Parameters						
^1^ FBG (mg/dL)	89 (15.5)	104 (26) ^a^	98 (36) ^a^	127 (49) ^ac^	<0.001	0.176
^1^ HbA1c (%)	5.4 (0.35)	5.9 (1.10) ^a^	6.3 (2.20) ^a^	7.1 (1.80) ^ab^	<0.001	0.250
^1^ HOMA-IR	1.4 (1.09)	3.3 (2.90) ^a^	5.5 (5.00) ^a^	7.6 (8.92) ^ab^	<0.001	0.496
^1^ Insulin(µIU/mL)	6.2 (4.8)	13.7 (9.8) ^a^	22.6 (16.6) ^a^	23.1(23.2) ^ab^	<0.001	0.455
^2^ TC (mg/dL)	194.9 ± 39.81	227.9 ± 38.55 ^a^	213.9 ± 40.3	189.9 ± 48.24 ^bc^	<0.001	0.119
^1^ TG (mg/dL)	68.5 (54.5)	162 (109) ^a^	166 (126) ^a^	138 (80) ^a^	<0.001	0.167
^1^ HDL-K (mg/dL)	59.5 (15.5)	48.2 (20.4) ^a^	48.6 (18.8) ^a^	50.8 (17.0) ^a^	0.001	0.080
^2^ LDL-C (mg/dL)	117.9 ± 33.81	139.5 ± 35.89 ^a^	130.3 ± 36.95	109.9 ± 40.28 ^b^	<0.001	0.088
^1^ ALT (U/L)	15 (11)	38 (37) ^a^	50 (42) ^a^	42 (49) ^a^	<0.001	0.207
^1^ AST (U/L)	19 (10)	26 (17) ^a^	41 (34) ^ab^	38 (29) ^ab^	<0.001	0.178
^1^ GGT (U/L)	16.5 (8)	32 (26) ^a^	37 (24) ^a^	68 (61) ^abc^	<0.001	0.196
^1^ Uric acid (mg/dL)	4.6 (1.9)	6 (2) ^a^	5.9 (2) ^a^	5.7 (1.8) ^a^	<0.001	0.053
^2^ Albumin (g/L)	45.6 ± 2.15	47.0 ± 2.75	45.0 ± 2.79 ^b^	44.2 ± 3.41 ^b^	<0.001	0.119
^2^ Lymphocytes (10^3^/µL)	1.8 ± 0.41	2.3 ± 0.71 ^a^	2.7 ± 0.73 ^ab^	2.1 ± 0.80 ^c^	<0.001	0.172
^2^ Platelets(10^3^/µL)	247.8 ± 47.04	255.0 ± 60.75	254.8 ± 57.41	196.9 ± 72.5 ^abc^	<0.001	0.142
^1^ DBil (mg/dL)	0.12 (0.07)	0.10 (0.06)	0.12 (0.07)	0.16 (0.10) ^b^	0.011	0.061
^1^ TBil (mg/dL)	0.64 (0.33)	0.64 (0.35)	0.65 (0.34)	0.70 (0.44)	0.647	0.009
^1^ INR	0.98 (0.06)	0.94 (0.09)	0.97 (0.06)	1.03 (0.13) ^bc^	<0.001	0.158
^1^ Ferritin (ng/mL)	28.05 (46.4)	49.8 (75.1) ^a^	67.9 (103.7) ^a^	71.8 (151.7) ^a^	<0.001	0.097
^1^ CRP (mg/L)	0.77 (1.6)	2.7 (2.7) ^a^	3.66 (4.61) ^a^	3.95 (6.25) ^ab^	<0.001	0.162
^2^ CAP (dB/m)	197.3 ± 23.6	308.9 ± 40.4 ^a^	312.9 ± 34 ^a^	293.8 ± 37.1 ^a^	<0.001	0.653

1: Kruskal–Wallis, 2: One Way ANOVA. F0: 25, F1: 22, F2: 47, F3: 19, F4: 26, C: 44; a: significant difference compared to the control group (*p* < 0.05), b: significant difference compared to the F0-F1 group (*p* < 0.05), c: significant difference compared to the F2 group (*p* < 0.05). FBG: fasting blood glucose, HOMA-IR: homeostatic model evaluation insulin resistance index, TC: total cholesterol, TG: triglyceride, HDL-C: high-density lipoprotein cholesterol, LDL-C: low-density lipoprotein cholesterol, ALT: alanine aminotransferase, AST: aspartate aminotransferase, GGT: gamma-glutamyl transferase, DBil: direct bilirubin, TBil: total bilirubin, INR: international normalized ratio, CRP: C-reactive protein, CAP: controlled attenuation parameter.

**Table 6:** Mean ($\bar{x}$), standard deviation (S), and median values of inflammation and oxidative stress markers of individuals by groups.

	Control	F0–F1	F2	F3–F4	*p* *	Effect Size
	(n = 44)	(n = 47)	(n = 47)	(n = 45)		
	$\bar{x}$± SD; Median (IQR)	$\bar{x}$± SD Median (IQR)	$\bar{x}$± SD Median (IQR)	$\bar{x}$± SD Median (IQR)		
Inflammation and oxidative stress markers						
TNF-α (pg/mL)	6.3 ± 1.21	7.8 ± 2.10	8.4 ± 1.74	9.7 ± 2.56 ^b^	0.020	0.057
IL-10 (pg/mL)	2.8 (1.05)	3 (1.25)	3.1 (0.84)	3.2 (1.85)	0.102	0.036
IL-6 (pg/mL)	2.1 (1.29)	2.3 (1.17)	2.7 (1.51)	3.6 (2.58) ^bc^	<0.001	0.121
GSH (mg/mL)	0.066 (0.025)	0.088 (0.057)	0.072 (0.051)	0.068 (0.038)	0.003	0.077
MDA (µmol/L)	3.3 (1.14)	4.5 (2.11) ^c^	3.0 (1.67)	3.7 (2.40)	0.001	0.087
MDA/GSH	49.3 (29.4)	47.1 (49.1)	47.5 (30.1)	54.8 (37.1)	0.877	0.004
TMAO (pg/mL)	53.1 (115.8)	42.1 (14.9)	47.6 (13.1)	38.8 (11.3)	0.014	0.060

*: These are *p*-values adjusted for age, BMI, sex, comorbidity, relevant medication use, MEDAS score, and physical activity. F0: 25, F1: 22, F2: 47, F3: 19, F4: 26, C: 44. b: Significant difference compared to the F0–F1-group (*p* < 0.05), c: Significant difference compared to the F2 group (*p* < 0.05); TNF-α: Tumor necrosis factor alpha, IL-10: Interleukin-10, IL-6: Interleukin-6, GSH: glutathione, MDA: malondialdehyde, MDA/GSH: malondialdehyde/glutathione ratio, TMAO: trimethylamine N-oxide.

**Table 7:** Distribution of MEDAS classification, individual items and total scores across groups.

	Control		F0–F1		F2		F3–F4		*p* *	Effect Size
	(n = 44)		(n = 47)		(n = 47)		(n = 45)			
	n	%	n	%	n	%	n	%		
MEDAS Classification										
Poor (0–6 points)	14	31.8	42	89.4 ^a^	43	91.5 ^a^	41	91.1 ^a^	<0.001	0.428
Moderate (7–8 points)	22	50	5	10.6 ^a^	4	8.5 ^a^	3	6.7 ^a^		
Good (9–14 points)	8	18.2	0	0 ^a^	0	0 ^a^	1	2.2 ^a^		
Individual MEDAS Items										
Olive oil as main fat	32	72.7	28	59.6	17	36.2 ^a^	15	33.3 ^a^	<0.001	0.326
Olive oil ≥ 4 ts/day	13	29.5	2	4.3 ^a^	2	4.3 ^a^	1	2.2 ^a^	<0.001	0.373
Vegetables ≥ 2 p/day	29	65.9	13	27.7 ^a^	6	12.8 ^a^	9	20 ^a^	<0.001	0.438
Fruits ≥ 3 p/day	23	52.3	12	25.5	15	31.9	8	17.8 ^a^	0.004	0.271
Red/processed meat < 1 p/day	34	77.3	34	72.3	35	74.5	34	75.6	0.958	0.041
Butter/margarine < 1 p/day	23	52.3	10	21.3 ^a^	12	25.5	11	24.4 ^a^	0.005	0.267
Sugary beverages < 1 p/day	31	70.5	26	55.3	17	36.2 ^a^	27	60	0.010	0.250
Red wine ≥ 7 p/week	1	2.3	0	0	0	0	0	0	0.240	0.132
Legumes ≥ 3 p/week	36	81.8	33	70.2	24	51.1 ^a^	20	44.4 ^a^	<0.001	0.304
Fish/seafood ≥ 3 p/week	7	15.9	5	10.6	0	0 ^a^	3	6.7	0.036	0.212
Pastry products < 3 times/week	27	61.4	20	42.6	15	31.9 ^a^	22	48.9	0.040	0.213
Nuts ≥ 3 p/week	40	90.9	28	59.6 ^a^	15	31.9 ^ab^	12	26.7 ^ab^	<0.001	0.506
Poultry products to red meat	21	47.7	19	40.4	20	42.6	17	37.8	0.807	0.073
Sofrito ≥ 2 times/week	20	45.5	8	17 ^a^	3	6.4 ^a^	1	2.2 ^a^	<0.001	0.438
MEDAS Total Score										
Median (IQR)	7.5 (3.0)		5.0 (2.0) ^a^		4.0 (1.0) ^ab^		4.0 (2.0) ^ab^		<0.001	0.442

*: Chi-square test or Kruskal–Wallis test, post hoc Dunn test with Bonferroni correction if required. F0: 25, F1: 22, F2: 47, F3: 19, F4: 26, C: 44; a: significant difference compared to the control group (*p* < 0.05), b: significant difference compared to the F0–F1 group (*p* < 0.05).

**Table 8:** Distribution of physical activity levels of individuals by groups.

	Control (n = 44)		F0–F1 (n = 47)		F2 (n = 47)		F3–F4 (n = 45)		*p* *	Effect Size
	Number	%	Number	%	Number	%	Number	%		
IPAQ-SF									0.004	0.229
Inactive (<600 MET)	19	43.2	36	76.6 ^a^	35	74.5 ^a^	34	75.6 ^a^		
Minimally active (600–3000 MET)	19	43.2	10	21.3	11	23.4	7	15.6 ^a^		
Very active (>3000 MET)	6	13.6	1	2.1	1	2.1	4	8.8		
IPAQ-SF score Median (IQR)	840 (1920)		270 (540) ^a^		225 (650) ^a^		90 (540) ^a^		<0.001	0.117

*: Chi-square test or Kruskal–Wallis test, post hoc Dunn test with Bonferroni correction if required. F0: 25, F1: 22, F2: 47, F3: 19, F4: 26, C: 44; a: significant difference compared to the control group (*p* < 0.05).

**Table 9:** Spearman correlation coefficients between variables.

Variables	1	2	3	4	5	6	7	8	9	10	11
1. CAP dB/m fat accumulation	—										
2. E kPa fibrosis	0.421 **	—									
3. TNF-α	0.343 **	0.547 **	—								
4. IL-10	0.106	0.298 **	0.242 **	—							
5. IL-6	0.183 *	0.439 **	0.349 **	0.286 **	—						
6. GSH	0.202 **	0.046	−0.047	0.159 *	0.045	—					
7. MDA	0.153 *	−0.005	−0.001	0.005	−0.052	0.111	—				
8. MDA/GSH ratio	−0.018	−0.042	0.012	−0.130	−0.074	—	—	—			
9. TMAO	−0.249 **	−0.278 **	−0.192 *	0.033	−0.100	−0.094	−0.067	0.009	—		
10. MEDAS total	−0.540 **	−0.567 **	−0.375 **	−0.111	−0.240 **	−0.202 **	0.051	0.168 *	0.168 *	—	
11. IPAQ-SF total	−0.170 *	−0.261 **	−0.186 *	−0.118	−0.100	−0.113	−0.231 **	−0.061	0.153 *	0.218 **	—

Note: Spearman rank difference correlation analysis was used. * *p* < 0.05. ** *p* < 0.001.

## Data Availability

The data that support the findings of this study will be made available by the corresponding author upon reasonable request. Data is not publicly available due to privacy and ethical concerns.

## Abbreviations

The following abbreviations are used in this manuscript:

MASLD	Metabolic Dysfunction-Associated Steatotic Liver Disease
MET	Metabolic Equivalent of Task
MD	Mediterranean Diet
TMA	Trimethylamine
TMAO	Trimethylamine N-oxide
IL-6	Interleukin-6
IL-10	Interleukin-10
TNF-α	Tumor Necrosis Factor-alpha
MDA	Malondialdehyde
GSH	Glutathione
MEDAS	Mediterranean Diet Adherence Screener
IPAQ-SF	International Physical Activity Questionnaire-Short Form
LSM	Liver Stiffness Measurement
CAP	Controlled Attenuation Parameter
UNESCO	United Nations Educational, Scientific and Cultural Organization
FMO3	Flavin-Containing Monooxygenase 3
MASH	Metabolic Dysfunction-Associated Steatohepatitis
ROS	Reactive Oxygen Species
EASL	European Association for the Study of the Liver
EASD	European Association for the Study of Diabetes
EASO	European Association for the Study of Obesity
BMI	Body Mass Index
T2DM	Type 2 Diabetes Mellitus
VCTE	Vibration-Controlled Transient Elastography
BIA	Bioelectrical Impedance Analysis

## References

[1] Rinella M.E., Lazarus J.V., Ratziu V., Francque S.M., Sanyal A.J., Kanwal F., Romero D., Abdelmalek M.F., Anstee Q.M., Arab J.P. (2023). A multisociety Delphi consensus statement on new fatty liver disease nomenclature. Hepatology.

[2] Sookoian S., Pirola C.J., Sanyal A.J. (2025). MASLD as a non-communicable disease. Nat. Rev. Gastroenterol. Hepatol..

[3] Targher G., Byrne C.D., Tilg H. (2024). MASLD: A systemic metabolic disorder with cardiovascular and malignant complications. Gut.

[4] Stefano J.T., Duarte S.M.B., Ribeiro Leite Altikes R.G., Oliveira C.P. (2023). Non-pharmacological management options for MAFLD: A practical guide. Ther. Adv. Endocrinol. Metab..

[5] Petroni M.L., Brodosi L., Bugianesi E., Marchesini G. (2021). Management of non-alcoholic fatty liver disease. BMJ.

[6] Dwidar A.M., Ferra A.I., Dwidar A.M., Esmail E.S., Elkafoury R.M. (2026). Metabolic features’ effect on FibroScan-AST (FAST) score in Egyptian patients with metabolic dysfunction-associated steatotic liver disease (MASLD). Egypt. Liver J..

[7] Lai J.C.-T., Liang L.Y., Wong G.L.-H. (2024). Noninvasive tests for liver fibrosis in 2024: Are there different scales for different diseases?. Gastroenterol. Rep..

[8] Keskin M., Arsoy H.A., Kara O., Sarandol E., Beyaz A., Koca N., Yilmaz Y. (2025). Metabolic and Hepatic Profiles of Non-Obese and Obese Metabolic Dysfunction-Associated Steatotic Liver Disease in Adolescents: The Role of FibroScan Parameters, Fibroblast Growth Factor-21, and Cytokeratin-18. Turk. J. Gastroenterol..

[9] Barr R.G., Ferraioli G., Palmeri M.L., Goodman Z.D., Garcia-Tsao G., Rubin J., Garra B., Myers R.P., Wilson S.R., Rubens D. (2015). Elastography assessment of liver fibrosis: Society of radiologists in ultrasound consensus conference statement. Radiology.

[10] Jalil S., Pagadala M., Dunn N., Blaney H., Elfeki M., Thakral N., Singal A.K. (2025). Transient Elastography and Fibroscan: Stethoscope of a Hepatologist in Today’s World. Curr. Hepatol. Rep..

[11] Romero-Gómez M., Zelber-Sagi S., Trenell M. (2017). Treatment of NAFLD with diet, physical activity and exercise. J. Hepatol..

[12] Hsieh M.L., Su T.H., Lin Y.C., Chen Y.Y., Tung C.F., Huang L.S., Wu C.H., Peng Y.C., Hsieh V.C.R. (2026). Mediterranean Diet Adherence Is Associated With Reduced Liver Fibrosis Risk in Metabolic Dysfunction–Associated Steatotic Liver Disease. J. Gastroenterol. Hepatol..

[13] Williams G.M., Hoedt E.C., Duncanson K., Gan L., Prakoso E., Talley N.J., Beck E.J. (2026). Inverse associations between Mediterranean diet constituents and the gut microbiota in metabolic-associated steatotic liver disease (MASLD): A case-control study. Nutr. Metab..

[14] Tekdemir S.N., Soykurt S.Ç. (2024). Popüler diyetlerin mikrobiyotaya etkisi. Int. J. Interdiscip. Interact. Health Sci..

[15] Rafie Sedaghat F., Ahmadi S., Samadpour Zahmat-dar T., Saedi S., Samadi Kafil H. (2026). The Effect of the Mediterranean Diet on Gut Microbiota and Its Impact on Neurodegenerative Diseases: A Narrative Review. Nutr. Diet. Suppl..

[16] Tacke F., Horn P., Wong V.W.-S., Ratziu V., Bugianesi E., Francque S., Zelber-Sagi S., Valenti L., Roden M., Schick F. (2024). EASL–EASD–EASO Clinical Practice Guidelines on the management of metabolic dysfunction-associated steatotic liver disease (MASLD). J. Hepatol..

[17] Öztürk F.M. (2005). Üniversitede Eğitim-Öğretim Gören Öğrencilerde Uluslararasi Fiziksel Aktivite Anketinin Geçerliliği ve Güvenirliği ve Fiziksel Aktivite Düzeylerinin Belirlenmesi. Master’s Thesis.

[18] Krishnan S., O’Connor L.E., Wang Y., Gertz E.R., Campbell W.W., Bennett B.J. (2022). Adopting a Mediterranean-style eating pattern with low, but not moderate, unprocessed, lean red meat intake reduces fasting serum trimethylamine N-oxide (TMAO) in adults who are overweight or obese. Br. J. Nutr..

[19] Huang Y., Zhang J., Zhang Y., Wang W., Li M., Chen B., Zhang X., Zhang Z., Huang J., Jin Y. (2024). Red meat intake, faecal microbiome, serum trimethylamine N-oxide and hepatic steatosis among Chinese adults. Liver Int..

[20] Theodoridis X., Papaemmanouil A., Papageorgiou N., Savopoulos C., Chourdakis M., Triantafyllou A. (2025). The association between lifestyle interventions and trimethylamine N-oxide: A systematic-narrative hybrid literature review. Nutrients.

[21] Chen W.-Y., Zhang J.-H., Chen L.-L., Byrne C.D., Targher G., Luo L., Ni Y., Zheng M.-H., Sun D.-Q. (2024). Bioactive metabolites: A clue to the link between MASLD and CKD?. Clin. Mol. Hepatol..

[22] Theofilis P., Vordoni A., Kalaitzidis R.G. (2022). Trimethylamine N-oxide levels in non-alcoholic fatty liver disease: A systematic review and meta-analysis. Metabolites.

[23] Termite F., Archilei S., D’Ambrosio F., Petrucci L., Viceconti N., Iaccarino R., Liguori A., Gasbarrini A., Miele L. (2025). Gut microbiota at the crossroad of hepatic oxidative stress and MASLD. Antioxidants.

[24] Hu Z., Yue H., Jiang N., Qiao L. (2025). Diet, oxidative stress and MAFLD: A mini review. Front. Nutr..

[25] Kim Y., Park Y., Rho H., Yao T., Gao B., Hwang S. (2025). Inflammation in MASLD progression and cancer. JHEP Rep..

[26] Wang L., Wang Y. (2026). Non-Invasive Tests for the Detection of MASLD: Biomarkers and Imaging for Staging Steatosis, MASH, and Fibrosis. Int. J. Gen. Med..

[27] Rinaldi L., Giorgione C., Mormone A., Esposito F., Rinaldi M., Berretta M., Marfella R., Romano C. (2023). Non-invasive measurement of hepatic fibrosis by transient elastography: A narrative review. Viruses.

[28] Sualeheen A., Tan S.Y., Georgousopoulou E., Daly R.M., Tierney A.C., Roberts S.K., George E.S. (2024). Mediterranean diet for the management of metabolic dysfunction-associated steatotic liver disease in non-Mediterranean, Western countries: What’s known and what’s needed?. Nutr. Bull..

[29] George E.S., Sualeheen A., Freer C., Georgeousopoulou E.N., Roberts S.K., Daly R.M., Tan S.-Y. (2026). Association between the Mediterranean dietary pattern and metabolic dysfunction-associated steatotic liver disease: A longitudinal analysis from UK BIOBANK. Br. J. Nutr..

[30] Elsabaawy M., Naguib M., Abuamer A., Shaban A. (2025). Comparative application of MAFLD and MASLD diagnostic criteria on NAFLD patients: Insights from a single-center cohort. Clin. Exp. Med..

[31] Pekcan G. (2008). Beslenme durumunun saptanmasi. Diyet El Kitabi.

[32] Sağlık Bakanlığı T.C. (2022). Türkiye Beslenme Rehberi (TÜBER). https://hsgm.saglik.gov.tr/depo/birimler/saglikli-beslenme-ve-hareketli-hayat-db/Dokumanlar/Rehberler/Turkiye_Beslenme_Rehber_TUBER_2022_min.pdf.

[33] Consultation W. (2008). Waist circumference and waist-hip ratio. Report of a WHO Expert Consultation.

[34] Pehlivanoğlu E.F.Ö., Balcıoğlu H., Ünlüoğlu İ. (2020). Akdeniz diyeti bağlılık ölçeği’nin türkçe’ye uyarlanması geçerlilik ve güvenilirliği. Osman. Tıp Derg..

[35] Ellman G.L. (1959). Tissue sulfhydryl groups. Arch. Biochem. Biophys..

[36] Draper H.H., Hadley M. (1990). Malondialdehyde determination as index of lipid Peroxidation. Methods Enzymol..

[37] Ohkawa H., Ohishi N., Yagi K. (1979). Assay for lipid peroxides in animal tissues by thiobarbituric acid reaction. Anal. Biochem..

[38] Suresh M.G., Mohamed S., Geetha H.S., Prabhu S., Trivedi N., Mehta P.D., Damodaran U.K., Brar A., Sohal A., Hatwal J. (2026). Cardiovascular implications in metabolic dysfunction-associated steatotic liver disease (MASLD): A state-of-the-art review. Korean Circ. J..

[39] Wang X., Han X., Liu J., Qi Y. (2026). The association of TyG-BMI with MAFLD and liver fibrosis: A cross-sectional study. Sci. Rep..

[40] Gordito Soler M., López-González Á.A., Vallejos D., Martínez-Almoyna Rifá E., Vicente-Herrero M.T., Ramírez-Manent J.I. (2024). Usefulness of body fat and visceral fat determined by bioimpedanciometry versus body mass index and waist circumference in predicting elevated values of different risk scales for non-alcoholic fatty liver disease. Nutrients.

[41] Julian M.T., Arteaga I., Toran-Monserrat P., Pera G., Pérez-Montes de Oca A., Ruiz-Rojano I., Casademunt-Gras E., Chacon C., Alonso N. (2024). The link between abdominal obesity indices and the progression of liver fibrosis: Insights from a population-based study. Nutrients.

[42] Cosma T., Avram L., Donca V., Grosu A., Stoicescu L., Buzdugan E., Nemes A., Balan A.-M., Crisan D. (2025). Refining MASLD Phenotypes: Clinical, Metabolic, and Elastographic Differences Between Adipose Tissue Dysfunction and Obesity-Driven Disease. Nutrients.

[43] Feng R.-N., Du S.-S., Wang C., Li Y.-C., Liu L.-Y., Guo F.-C., Sun C.-H. (2014). Lean-non-alcoholic fatty liver disease increases risk for metabolic disorders in a normal weight Chinese population. World J. Gastroenterol. WJG.

[44] Nguyen L.N.B., Vo T.D. (2025). Comparison of Triglyceride-Glucose Index Indices and Fatty Liver Index in Predicting Metabolic Dysfunction-Associated Fatty Liver Disease: A Cross-Sectional Study Conducted in Vietnam. Life.

[45] Bae M., Kim K.M., Jin M.H., Yoon J.-H. (2025). Synergistic impact of serum uric acid and ferritin on MAFLD risk: A comprehensive cohort analysis. Sci. Rep..

[46] Wu Y., Zheng G., Zhang F., Li W. (2025). Association of high-sensitivity C-reactive protein with hepatic fibrosis in patients with metabolic dysfunction-associated steatotic liver disease. Front. Immunol..

[47] Guo W., Lin F., Yu C., Lu J., Qin P., Zhao X., Li X., Zhang Q. (2026). Serum lipoprotein (a) levels are inversely associated with metabolic dysfunction-associated steatosis liver disease progression: Two cross-sectional studies and a longitudinal study. Front. Nutr..

[48] Miura K., Arai N., Goka R., Morimoto N., Watanabe S., Isoda N., Yamamoto H., Kotani K. (2021). Oxidized high-density lipoprotein shows a stepwise increase as fibrosis progresses in patients with nonalcoholic fatty liver disease. Antioxidants.

[49] Martínez-Sánchez F.D., Corredor-Nassar M.J., Feria-Agudelo S.M., Paz-Zarza V.M., Martinez-Perez C., Diaz-Jarquin A., Manzo-Santana F., Sánchez-Gómez V.A., Rosales-Padron A., Baca-García M. (2025). Factors associated with advanced liver fibrosis in a population with type 2 diabetes: A multicentric study in Mexico City. J. Clin. Exp. Hepatol..

[50] Commins I., Clayton-Chubb D., Janko N., Majeed A., Kemp W., Roberts S. (2026). Efficacy and Safety of Statins in MASLD and Other Chronic Liver Diseases. Med. Sci..

[51] Jamialahmadi T., Reiner Ž., Riahi M.M., Kesharwani P., Eid A.H., Tayarani-Najaran Z., Sahebkar A. (2025). The effects of statin therapy on circulating levels of trimethylamine N-oxide: A systematic review and meta-analysis. Curr. Med. Chem..

[52] Canyelles M., Borràs C., Rotllan N., Tondo M., Escolà-Gil J.C., Blanco-Vaca F. (2023). Gut microbiota-derived TMAO: A causal factor promoting atherosclerotic cardiovascular disease?. Int. J. Mol. Sci..

[53] Bürki J.T., Schropp J., Neyer P., Steuer C., Bosch J., Berzigotti A., Rodrigues S.G. (2025). Exploring the trimethylamine pathway in advanced chronic liver disease. npj Gut Liver.

[54] Duan Y., Pan X., Luo J., Xiao X., Li J., Bestman P.L., Luo M. (2022). Association of inflammatory cytokines with non-alcoholic fatty liver disease. Front. Immunol..

[55] Eid R.A., Hamed A.M., Elgendy S.O., Orayj K.M., Ibrahim A.R., Hamied A.M.A., Wahsh E.A., Youssif M., Rabea H., Madney Y.M. (2025). Associations Between Systemic Inflammatory Markers, Metabolic Dysfunction, and Liver Fibrosis Scores in Patients with MASLD. Metabolites.

[56] Taru V., Szabo G., Mehal W., Reiberger T. (2024). Inflammasomes in chronic liver disease: Hepatic injury, fibrosis progression and systemic inflammation. J. Hepatol..

[57] Potoupni V., Georgiadou M., Chatzigriva E., Polychronidou G., Markou E., Zapantis Gakis C., Filimidou I., Karagianni M., Anastasilakis D., Evripidou K. (2021). Circulating tumor necrosis factor-α levels in non-alcoholic fatty liver disease: A systematic review and a meta-analysis. J. Gastroenterol. Hepatol..

[58] Vachliotis I.D., Polyzos S.A. (2025). The intriguing roles of cytokines in metabolic dysfunction-associated steatotic liver disease: A narrative review. Curr. Obes. Rep..

[59] Bocsan I.C., Milaciu M.V., Pop R.M., Vesa S.C., Ciumarnean L., Matei D.M., Buzoianu A.D. (2017). Cytokines Genotype-Phenotype Correlation in Nonalcoholic Steatohepatitis. Oxidative Med. Cell. Longev..

[60] Marques P., Francisco V., Martinez-Arenas L., Carvalho-Gomes A., Domingo E., Piqueras L., Berenguer M., Sanz M.-J. (2023). Overview of cellular and soluble mediators in systemic inflammation associated with non-alcoholic fatty liver disease. Int. J. Mol. Sci..

[61] Xu H.-L., Wan S.-R., An Y., Wu Q., Xing Y.-H., Deng C.-H., Zhang P.-P., Long Y., Xu B.-T., Jiang Z.-Z. (2024). Targeting cell death in NAFLD: Mechanisms and targeted therapies. Cell Death Discov..

[62] Köroğlu E., Canbakan B., Atay K., Hatemi İ., Tuncer M., Dobrucalı A., Sonsuz A., Gültepe İ., Şentürk H. (2016). Role of oxidative stress and insulin resistance in disease severity of non-alcoholic fatty liver disease. Turk. J. Gastroenterol..

[63] Svobodová G., Horní M., Velecká E., Boušová I. (2025). Metabolic dysfunction-associated steatotic liver disease-induced changes in the antioxidant system: A review. Arch. Toxicol..

[64] Lee S.W., Huang D.Q., Bettencourt R., Ajmera V., Tincopa M., Noureddin N., Amangurbanova M., Siddiqi H., Madamba E., Majzoub A.M. (2024). Low liver fat in non-alcoholic steatohepatitis-related significant fibrosis and cirrhosis is associated with hepatocellular carcinoma, decompensation and mortality. Aliment. Pharmacol. Ther..

[65] Liu W.-Y., Huang S., Ji H., Kim S.U., Yip T.C.-F., Wong G.L.-H., Petta S., Tsochatzis E., Nakajima A., Bugianesi E. (2025). From “Burnt-Out” to “Burning-Out”: Capturing Liver Fat Loss in Patients With Advanced Metabolic Dysfunction–Associated Steatotic Liver Disease from a Dynamic Perspective. Gastroenterology.

[66] Pădureanu V., Boldeanu L., Pîrșcoveanu D.F.V., Dop D., Cioboată R., Bobîrcă A., Rădulescu V.M. (2025). Oxidative Stress and Cirrhosis Severity: A Retrospective Cohort Analysis of Predictive and Interactive Effects with Inflammation. Metabolites.

[67] Li X.S., Obeid S., Klingenberg R., Gencer B., Mach F., Räber L., Windecker S., Rodondi N., Nanchen D., Muller O. (2017). Gut microbiota-dependent trimethylamine N-oxide in acute coronary syndromes: A prognostic marker for incident cardiovascular events beyond traditional risk factors. Eur. Heart J..

[68] Evans M., Dai L., Avesani C.M., Kublickiene K., Stenvinkel P. (2023). The dietary source of trimethylamine N-oxide and clinical outcomes: An unexpected liaison. Clin. Kidney J..

[69] Deniz M.Ş., Baş M. (2025). Short-Term Mediterranean Dietary Intervention Reduces Plasma Trimethylamine-N-Oxide Levels in Healthy Individuals. Nutrients.

[70] van den Berg E.H., Flores-Guerrero J.L., Garcia E., Connelly M.A., de Meijer V.E., Investigators T., Bakker S.J., Blokzijl H., Dullaart R.P. (2023). High plasma levels of betaine, a trimethylamine N-Oxide-related metabolite, are associated with the severity of cirrhosis. Liver Int..

[71] Alzughayyar D.-K.N., Weber R.-M., Husain S., Schoch N., Englert H. (2025). Impact of the Healthy Lifestyle Community Program (HLCP-3) on Trimethylamine N-Oxide (TMAO) and Risk Profile Parameters Related to Lifestyle Diseases During the Six Months Following an Intervention Study. Nutrients.

[72] Randrianarisoa E., Lehn-Stefan A., Wang X., Hoene M., Peter A., Heinzmann S.S., Zhao X., Königsrainer I., Königsrainer A., Balletshofer B. (2016). Relationship of serum trimethylamine N-oxide (TMAO) levels with early atherosclerosis in humans. Sci. Rep..

[73] Costabile G., Vetrani C., Bozzetto L., Giacco R., Bresciani L., Del Rio D., Vitale M., Della Pepa G., Brighenti F., Riccardi G. (2021). Plasma TMAO increase after healthy diets: Results from 2 randomized controlled trials with dietary fish, polyphenols, and whole-grain cereals. Am. J. Clin. Nutr..

[74] Barrea L., Annunziata G., Muscogiuri G., Laudisio D., Di Somma C., Maisto M., Tenore G.C., Colao A., Savastano S. (2019). Trimethylamine N-oxide, Mediterranean diet, and nutrition in healthy, normal-weight adults: Also a matter of sex?. Nutrition.

[75] Vich Vila A., Collij V., Sanna S., Sinha T., Imhann F., Bourgonje A.R., Mujagic Z., Jonkers D.M., Masclee A.A., Fu J. (2020). Impact of commonly used drugs on the composition and metabolic function of the gut microbiota. Nat. Commun..

[76] Li D.Y., Li X.S., Chaikijurajai T., Li L., Wang Z., Hazen S.L., Tang W.W. (2022). Relation of statin use to gut microbial trimethylamine N-oxide and cardiovascular risk. Am. J. Cardiol..

[77] Su C., Li X., Yang Y., Du Y., Zhang X., Wang L., Hong B. (2021). Metformin alleviates choline diet-induced TMAO elevation in C57BL/6J mice by influencing gut-microbiota composition and functionality. Nutr. Diabetes.

[78] Kuka J., Videja M., Makrecka-Kuka M., Liepins J., Grinberga S., Sevostjanovs E., Vilks K., Liepinsh E., Dambrova M. (2020). Metformin decreases bacterial trimethylamine production and trimethylamine N-oxide levels in db/db mice. Sci. Rep..

[79] Barrea L., Verde L., Savastano S., Colao A., Muscogiuri G. (2023). Adherence to Mediterranean diet: Any association with NAFLD?. Antioxidants.

[80] Cano-Lallave L., Ruiz-Tovar J., Martin-de-Bernardo L., Martinez-Oribe M., Rodriguez-Obispo C., Carrascosa-Corrochano S., Martín-Nieto A., Baeza I., Gonzalez-Ramos M., Benito M. (2024). Influence of Adherence to the Mediterranean Diet and Level of Physical Activity with Liver Steatosis in People Aged > 50 Years and with a BMI > 25 kg/m^2^: Association with Biochemical Markers. Nutrients.

[81] Perez-Diaz-del-Campo N., Rosso C., Caviglia G., Castelnuovo G., D’Amato D., Abdulle A., Guariglia M., Armandi A., Olivero A., Abate M. (2023). Low adherence to Mediterranean Diet is associated to sCD163 levels in patients with MAFLD. Dig. Liver Dis..

[82] Mokhtare M., Abdi A., Sadeghian A.M., Sotoudeheian M., Namazi A., Sikaroudi M.K. (2023). Investigation about the correlation between the severity of metabolic-associated fatty liver disease and adherence to the Mediterranean diet. Clin. Nutr. ESPEN.

[83] Lee J.Y., Kim S., Lee Y., Kwon Y.-J., Lee J.-W. (2024). Higher adherence to the Mediterranean diet is associated with a lower risk of Steatotic, alcohol-related, and metabolic dysfunction-associated Steatotic liver disease: A retrospective analysis. Nutrients.

[84] Bianco A., Bonfiglio C., Franco I., Bagnato C.B., Verrelli N., Stabile D., Shahini E. (2025). Sedentary Behavior as a Risk Factor for Liver Fibrosis Development in Patients with Metabolic Dysfunction-Associated Steatotic Liver Disease (MASLD): A Cross-Sectional Study. J. Clin. Med..

[85] Lotfi A., Saneei P., Hekmatdost A., Salehisahlabadi A., Shiranian A., Ghiasvand R. (2019). The relationship between dietary antioxidant intake and physical activity rate with nonalcoholic fatty liver disease (NAFLD): A case–Control study. Clin. Nutr. ESPEN.

[86] Bagnato C.B., Bianco A., Bonfiglio C., Franco I., Verrelli N., Carella N., Shahini E., Zappimbulso M., Giannuzzi V., Pesole P.L. (2024). Healthy lifestyle changes improve cortisol levels and liver steatosis in MASLD patients: Results from a randomized clinical trial. Nutrients.

